# Supplementary material for: GSuite HyperBrowser: integrative analysis of dataset collections across the genome and epigenome
Source: Gigascience. 2017 Apr 27;6(7):1–12. doi: 10.1093/gigascience/gix032 (PMC5493745; doi:10.1093/gigascience/gix032)
Supplement: Additional File 1 — A text document describing statistical measures and hypothesis tests for suites of genomic tracks. The document contains detailed formulas and algorithms for statistical methodology used by the GSuite HyperBrowser system (PDF format, 13 806 KB). [file gix032_Additional_file_1.pdf]

Additional file 1:
Statistical measures and hypothesis tests for suites of genomic tracks

Simovski et al., "GSuite HyperBrowser:
integrative analysis of dataset collections across the genome and epigenome"

In this Supplementary Material we describe statistics and tests for suites of tracks for five different groups of questions. The first section describes the statistics that are common to several of the questions. It is followed by a section presenting the simulation algorithms common to several statistical tests while the next five sections describe the statistics and tests for one group of questions each. The last section gives an overview of the correspondence between terminology used in this supplementary and in the web interface of the GSuite HyperBrowser system. This text is more general than what is included in the GSuite HyperBrowser system at present but it include all implemented options.

The five questions concern:

1. Which tracks (in a suite) are most representative and most atypical?
2. Which tracks (in a suite) coincide most strongly with a separate single track?
3. Are certain tracks of one suite coinciding particularly strongly with certain tracks of another suite?
4. Which genomic regions are the most enriched with points/segments of the tracks of the suite?
5. In which genomic regions are the tracks of the suite coinciding the most?

In this supplementary note we formulate all text for segment tracks, but all text, i.e. questions, statistics, formulas and algorithms work equally well for point tracks. Point tracks are often easier since points either overlap completely or do not overlap at all. This is in contrast to segments that may overlap partly. When we divide into bins, there are typically between 50 and 1.000 bins. A typical dataset consists of several suites of tracks with 10-1000 tracks in each suite. In most tracks there are many segments and the characteristics we are looking for may be represented in 1.000-100.000 segments and even more per track.

Test statistics

This section describes test statistics that are common for several of the tests. We only describe test statistics for the entire track. It is possible to use the same test statistics for each bin separately. We prefer to describe this in the text instead of introducing additional indexes for the variables.

We use the following notation: $\mathrm{bp}(A_i)$ is the number of base pairs inside segments in the track A_i; $A_i \cup A_j$ is the track with segments in positions where at least one of the tracks A_i and A_j have a segment; $A_i \cap A_j$ is the track with segment in positions where the two tracks A_i and A_j have

overlapping segments. N is number of base pairs. We define several test statistics for comparing two tracks:

- $T_1(A_i, A_j) = \frac{bp(A_i \cap A_j)}{bp(A_i)}$ is non-symmetric and finds the ratio of segments in A_i that are also in A_j.
- $T_2(A_i, A_j) = \frac{bp(A_j)}{bp(A_i \cup A_j)}$ is non-symmetric and finds the ratio of A_j segments inside $A_i \cup A_j$ segments.
- $T_3(A_i, A_j) = \frac{bp(A_i \cap A_j)}{bp(A_i \cup A_j)}$ is symmetric and finds the ratio of overlapping segments relative to the union of segments, i.e. the probability for being inside a segment in both tracks when being inside a segment in at least one of the tracks. This is known as the Jaccard index [1].
- $T_4(A_i, A_j) = \frac{bp(A_i \cap A_j)/N}{\sqrt{(bp(A_i)/N)\ (bp(A_j)/N)}} = \frac{bp(A_i \cap A_j)}{\sqrt{bp(A_i) bp(A_j)}}$ is symmetric and is the ratio between the probability of being inside segments in the intersection of two tracks and the geometric mean of the probabilities of being inside segments in one of the two tracks.
- $T_5(A_i, A_j) = \frac{bp(A_i \cap A_j)/N}{(bp(A_i)/N)\ (bp(A_j)/N)} = \frac{N bp(A_i \cap A_j)}{bp(A_i)\ bp(A_j)}$ is symmetric and is the ratio between the probability of being inside segments in the intersection of two tracks and the product of the probabilities of being inside segments in one of the two tracks. This statistic is known as the Forbes coefficient defined in 1842/1907 [2]. The expected value of $T_5(A_i, A_j)$ is equal to 1 if the two tracks are independent, independently of the coverage of the segments in the two tracks. In the Appendix, we show that $Var(\ T_5(A_i, A_j)) \approx N(Var(Z) + 2E(Z)^2)/(bp(A_i) bp(A_j) E(L))$ where Z is the length of an overlap between two segments, given that there is an overlap, and $E(L)$ is the average length of a segment. The expectation and variance of Z are found from the distribution of the segment lengths in the two tracks. The formula for variance assumes that $(bp(A_i)/N)$ and $(bp(A_j)/N)$ are small. Notice that the standard deviation depends heavily on the total length of the segments. Therefore, it may be more robust to use statistic T_6 defined below.
- $T_6(A_i, A_j) = \frac{bp(A_i \cap A_j)/N - (bp(A_i)/N)\ (bp(A_j)/N)}{\sigma(bp(A_i \cap A_j)/N\ )}$ where $Var\big(bp(A_i \cap A_j)/N\big) \approx\ bp(A_i) bp(A_j)(Var(Z) + 2E(Z)^2)/(N^3 E(L))$. The expected value of T_6 is 0 and the variance is approximately 1. If the length distribution of the segments does not vary too much between the tracks, then the formulas for the expected value and variance are sufficiently good.
- $T_7(A_i, A_j, A_{-i}) = \frac{T_5(A_i, A_j)}{\frac{1}{n-1} \sum_{k \neq i} T_5(A_i, A_k)}$. This is a normalization of T_5 in respect to the reference GSuite. This may be denoted as a normalized Forbes coefficient.
- $T_8(A_i, A_j, b) = cov\left(X_b(A_i), X_b\big(A_j\big)\right)$ where $X_b(A)$ is a vector with length equal to number of bins in b where each element is the average coverage of segments from track A in the bin.

We have $0 \leq T_1, T_2, T_3, T_4 \leq 1$. Based on the statistics for comparing two tracks, we will define test statistics for each track based on track A_i's interaction with each of the other tracks in the suite of tracks $A = \{\ A_1, \dots, A_n\}$:

- $Q_{k,1}(A_i, A_{-i}) = min_{j \neq i}(T_k(A_i, A_j))$ finds the minimum interaction with the other tracks.
- $Q_{k,2}(A_i, A_{-i}) = max_{j \neq i}(T_k(A_i, A_j))$ finds the maximum interaction with the other tracks.
- $Q_{k,3}(A_i, A_{-i}) = \frac{1}{n-1}\sum_{j \neq i} T_k(A_i, A_j)$ finds the average interaction with the other tracks.
- $Q_{k,4}(A_i, A_{-i}) = min_{j \neq i}(T_k(A_j, A_i))$ finds the minimum interaction with the other tracks.
- $Q_{k,5}(A_i, A_{-i}) = max_{j \neq i}(T_k(A_j, A_i))$ finds the maximum interaction with the other tracks.
- $Q_{k,6}(A_i, A_{-i}) = \frac{1}{n-1}\sum_{j \neq i} T_k(A_j, A_i)$ finds the average interaction with the other tracks.

In the formulas above A_{-i} is the suite of tracks A except the track A_i. We need the three last alternatives for k=1,2 since $T_1(A_i, A_j)$ and $T_2(A_i, A_j)$ are not symmetric. We may use the test statistics defined above and further define properties of a suite:

- $R_{k,s,1}(A) = min_i(Q_{k,s}(A_i, A_{-i}))$ finds the minimum interaction between tracks.
- $R_{k,s,2}(A) = max_i(Q_{k,s}(A_i, A_{-i}))$ finds the maximum interaction between tracks.
- $R_{k,s,3}(A) = \frac{1}{n}\sum_i Q_{k,s}(A_i, A_{-i})$ finds the average interaction between tracks.
- $S_i\ (A)$ is the ratio of the base pairs that are inside exactly *i* segments from the suite A, $i = 1, \ldots, n$, where n is the number of tracks in A.

$R_{k,s,u}(A)$ measures how similar the tracks are in the suite A, while $S_i(A)$ measures how overlapping the segments are for the different tracks in the suite A.

The statistic $S_i(A)$ is used for identifying whether some of the segments are significantly frequent/rare in the suite. $S_i(A)$ may be use in illustrations in tables or plots or in descriptive data analysis and as a test statistic in a hypothesis test.

It is also possible to define statistics comparing two suites A and B:

- $U_{k,1}(A, B) = min_{i,j}(T_k(A_i, A_j))$ finds the minimum interaction between the tracks from each suite.
- $U_{k,2}(A, B) = max_{i,j}(T_k(A_i, A_j))$ finds the maximum interaction between the tracks from each suite.
- $U_{k,3}(A, B) = \frac{1}{nm}\sum_{i,j} T_k(A_i, A_j)$ finds the average interaction between the tracks from each suite.

Simulation algorithms

All the simulation algorithms below are based on randomizing segments and intersegments within the tracks in the suite. There are many different alternatives and only some of these are described here. We define three groups of algorithms that randomize: i) track separately, ii) between tracks and iii) tracks from several suites of the same type.

Algorithms that treat each track separately:

- **Track simulation segment-intersegment:** Independent simulation of each track by randomizing the order of segments and the order of intersegments.

Algorithms that randomize between tracks from a suite:

- **Suite simulation uniform:** Randomizing the order of segments and the order of intersegments from all the tracks. The tracks are simulated one by one by drawing successively a segment and then an intersegment from all the tracks without replacement. The segment/intersegment at the end of each track is split in two and continued at the start of the next track.
- **Suite simulation proportional:** Randomizing the order of segments and the order of intersegments from all the tracks. The tracks are simulated one by one by drawing successively a segment and then an intersegment from all the tracks without replacement. The drawing is not uniform, but biased in order to respect the average length distribution of the segments and intersegments of the track. The segment/intersegment at the end of each track is split in two and continued at the start of the next track.
- **Suite simulation uniform, fixed position:** Segments are randomized between tracks such that the position is fixed, but randomized between the tracks. The largest segments are simulated first and then in decreasing order. Overlap is not allowed. Starting with no segments and then adding segments in decreasing order at random and uniformly between the tracks.
- **Suite simulation proportional, fixed position:** Segments are randomized between tracks such that the position is fixed but randomized between the tracks. The largest segments are simulated first and then in decreasing order. Overlap is not allowed. Starting with no segments and then adding segments in decreasing order at random and proportional with $\mathrm{bp}(A_i)$ between the tracks.

If there are many segments, the algorithms with fixed positions may give very little variability. It is possible to make examples where it is not possible to change segments between tracks. In this case, it is necessary to use the other simulation algorithms.

Algorithms that randomize tracks from several suites of same type:

- **Two suite simulation fixed position:** Segments are randomized between tracks of the two suites such that the position is fixed, but randomized between the tracks. The largest segments are simulated first and then in decreasing order. Overlap is not allowed. Starting with no segments and then adding segments in decreasing order at random proportional with $\mathrm{bp}(A_i)$ between the tracks.
- **Two suite simulation, randomize tracks:** The tracks are randomized between the suites.

The five questions

1. Which tracks (in a suite) are most representative and most atypical?

Compare tracks $A = A_1, \ldots, A_n$ with segments in a suite of tracks.

Descriptive statistics for ranking

We define the most representative track of the suite to be the track with the largest $Q_{k,s}(A_i, A_{-i})$. It is possible to argue for several different alternatives choices of $Q_{k,s}(A_i, A_{-i})$. However, $Q_{5,3}(A_i, A_{-i})$, or $Q_{3,3}(A_i, A_{-i})$ may be natural choices since they find the average interaction between track A_i and one other track in the suite, and are best on often used pairwise similarity measures at the core. The

interaction between two tracks is defined as the overlap between segments in the tracks relative to the union of the segments in the tracks. $Q_{1,1}(A_i, A_{-i})$ is largest for a track A_i that only has segments inside the segments of the other tracks. $Q_{1,4}(A_i, A_{-i})$ is largest for a track A_i that has segments that include most of the segments for all the other tracks. $Q_{1,2}(A_i, A_{-i})$ is largest for a track A_i that only has segments inside the segments of at least one of the other tracks. $Q_{1,5}(A_i, A_{-i})$ is largest for a track A_i that has segments that include the segments of at least one of the other tracks. These properties makes the two last alternatives less relevant if we do not want to focus on finding two tracks that are very similar.

Similarly, we define the most atypical track as the track in the suite with the smallest $Q_{k,s}(A_i, A_{-i})$.

Hypothesis testing per track

Question
Is the most/least typical track more/less typical than one would expect any of the tracks to be by chance?

Test statistics
We may use the same test statistics as the statistics in the descriptive ranking.

Null model
Each track is independent of the rest of the suite. Note that the different simulation algorithms define different populations of tracks which may be important to the result of the hypothesis testing since the simulation algorithm defines the interpretation of independent.

Simulation algorithm
Several different simulation algorithms may be used including *track simulation segment-intersegment, suite simulation uniform, suite simulation proportional, suite simulation uniform fixed position and suite simulation proportional fixed position.* The simulation algorithms will generate several suites. For each suite, we find the largest, second largest, etc. of $Q_{k,s}(A_i, A_{-i})$ for $i = 1, \ldots, n$. From the set of suites, we find the distribution of each of these ordered variables. The hypothesis is rejected iff the observed largest $Q_{k,s}(A_i, A_{-i})$ is larger than the α-quantile in the distribution for the largest $Q_{k,s}$. Similarly, we may focus on the smallest values in order to find the most atypical.

Hypothesis testing on full suite

Question
Are the tracks in the suite (as a whole) more/less similar than expected by chance?

Test statistic
It is possible to argue for several different alternatives to measure the similarity of tracks. However, it is most natural to use $R_{3,3,3}(A)$ as it measures the average interaction with all the other tracks in the suite. An alternative is $R_{k,s,2}(A)$ for $s = 2$ or $s = 5$ that finds the interaction to the track that has the largest interaction to the remaining tracks or $R_{6,3,3}(A)$.

Note that this is very similar to find the most representative track. The difference is that the test statistic for the most representative track focuses on finding tracks that are different. Hence, one track that is different from the others may result in a rejection of the null hypothesis. Here, we focus

on common properties of the entire suite. The test statistic $S_i(A)$ is used for identifying whether some of the segments are significantly frequent/rare in the suite.

Null model and simulation algorithm

The null model and simulation algorithm may be the same as when we have a hypothesis for each track.

It is possible to make a more efficient simulation algorithm dedicated to the test statistic $S_i(A)$, but this is probably not necessary. It may be of value to compare the observed statistic $S_i(A)$ relative to the corresponding average/mean of the distribution in addition to more extreme quantiles and illustrate this in a plot.

2. Which tracks (in a suite) coincide most strongly with a separate single track?

Compare track A_0 with segments to the suite B of tracks $B_1, \dots, B_n$ with segments.

Descriptive statistics for ranking

Here we want to rank the tracks in B on how much they coincide with A_0. It is natural to use the statistic $T_k(A_i, A_j)$. Natural choices may be to use the Forbes coefficient $T_5(A_i, A_j)$, the normalized Forbes coefficient $T_7(A_i, A_j, A_{-i})$ or the Jaccard index $T_3(A_i, A_j)$.

Hypothesis testing per track

Question

Is a track B_i from the suite B co-occurring more with the track A_0 than a track from the suite $B$is expected to do by chance?

This question is for each track in the suite and may require controlling the false discovery rate, FDR. Alternatively, we may also ask whether the most co-occurring track in the suite co-occur more than expected by chance.

Test statistics

We may use the same test statistics as the statistics in the descriptive ranking.

Null model

The track A_0 and all the tracks $B_1, \dots, B_n$ in the suite B are independent of the other tracks. As different simulation algorithms define different populations of tracks that again lead to different interpretations of independent, we describe different simulation methods below.

Simulation algorithm

We may either simulate the track A_0 or the tracks in the suite B. The advantage of simulating A_0 is that this maintains the correlation structure in the suite. Track A_0 may be simulated by *Track simulation segment-intersegment* or other simulation algorithms for single tracks. The suite may be simulated by *suite simulation uniform, suite simulation proportional, suite simulation uniform fixed position, suite simulation proportional fixed position* and also other suite simulation algorithms. These simulation algorithms may simulate many more tracks than there are tracks in the suite. All these tracks are based on the segments in the tracks in the suite.

By simulating a large number of possible tracks from the suite, we may find the null distribution, i.e. the distribution for the test statistics making it possible to find a p-value.

Hypothesis testing on full suite

Question

Do the tracks in the suite (as a whole) coincide more/less with the separate single track A_0 than expected by chance?

Test statistics

It is natural to use the statistic $Q_{k,s}(A_0, B)$. A natural choice may be $Q_{5,3}(A_0, B)$ that is based on average interaction between track A_0 and one track in the suite, but in special situations it is possible to argue for other test statistics. The interaction between two tracks is defined as the overlap between segments in the tracks relative to the union of the segments in the tracks. $Q_{1,1}(A_0, B)$ is the minimum overlap of the segments in the tracks in B with the segments in track A_0. $Q_{1,1}(A_0, B)$ is largest for a track A_0 that only has segments inside the segments of tracks in B. $Q_{1,4}(A_0, B)$ is largest for a track A_0 that has segments that include most of the segments for all the other tracks. $Q_{1,2}(A_0, B)$ is largest for a track A_i that only has segments inside the segments of at least one of the other tracks. $Q_{1,5}(A_0, B)$ is largest for a track A_0 that has segments that include the segments of at least one of the other tracks.

Null model and simulation algorithm

The null model and simulation algorithm may be the same as when we have a hypothesis for each track.

3. Are certain tracks of one suite coinciding particularly strongly with certain tracks of another suite?

Compare a suite of tracks $A = A_1, \dots, A_n$ with segments to a suite of tracks $B = B_1, \dots, B_m$ with segments.

Descriptive statistics for each pairwise combination of tracks

Here we want to rank each pairwise combination of track A_i from A and track B_j from B. It is natural to use the statistic $T_k(A_i, B_j)$. Natural choices may be to use the Forbes coefficient $T_5(A_i, B_j)$, the normalized Forbes coefficient $T_7(A_i, B_j, A_{-i})$ or the Jaccard index $T_3(A_i, B_j)$.

When the tracks in B consist of points, we can use the method developed in Sandve et al. [3]. It was illustrated with segment tracks for diseases and point tracks for transcription factor (TF) binding sites. Here we used two different z-score test statistics that both were normalized to expected value 0 and variance 1. In the main scheme, the null-hypothesis is that the proportion of binding sites associated to a given TF is the same within the regions of a given disease as it is across all diseases. The first test further assumes in the null hypothesis that binding locations of a given TF falls uniformly among the set of positions containing binding locations for any TF. In the alternative scheme, the null hypothesis is that binding locations of the given TF falls inside gene regions of the given disease proportionally to how often the binding locations of this TF on average falls inside gene region sets across all diseases. The first test is based on the hypergeometric distribution, while the second test is based on the binomial distribution.

Hypothesis testing per track

Question

Is a track from one suite co-occurring more with a track from the other suite than expected by chance (given the general propensity of each of the two tracks to co-occur with tracks of the other suite)?

This question is for each pair of tracks and may require controlling the false discovery rate, FDR. Alternatively, we may also ask whether the most co-occurring pair of tracks from each of the two suites co-occur more than expected by chance.

Test statistic

It is natural to use the statistic $T_k(A_i, B_j)$. Natural choices may be to use the Forbes coefficient $T_5(A_i, B_j)$, the normalized Forbes coefficient $T_7(A_i, B_j, A_{-i})$ or the Jaccard index $T_6(A_i, B_j)$.

Null model

The tracks of each suite are independent of the other tracks in the suite and of the tracks in the other suite. As different simulation algorithms define different populations of tracks that again lead to different interpretations of independent, we describe different simulation methods below.

The null hypothesis must assume certain statistical properties of the two suites that either are the same or that are different for the two suites.

Simulation algorithm

It is natural to simulate one of the suites and keep the other fixed. We should probably fix the suite where it is most important to keep the correlation structure in the suite.

Several different simulation algorithms may be used including *track simulation segment-intersegment, suite simulation uniform, suite simulation proportional, suite simulation uniform fixed position* and *suite simulation proportional fixed position.* As usual, we calculate the test statistic for each simulation to find an estimate of the null distribution.

Hypothesis testing on full suite

Question

Are the tracks from one suite co-occuring more with a track from the second suite than expected by chance (given the general propensity of each of the two tracks to co-occur with tracks of the other suite)?

Test statistic

It is natural to use the statistic $U_{k,s}(A, B)$ comparing the two suites.

Null model and simulation algorithm

The null model and simulation algorithm may be the same as when we have a hypothesis for each track.

4. Which genomic regions are the most enriched with segments of the tracks of the suite?

Compare a suite of tracks $A = A_1, \dots, A_n$ with segments in a sequence of bins $b_1, \dots, b_k$.

Descriptive statistics for ranking

Here we want to rank the bins based on the occurrence of elements of the tracks in the suite. It is most natural to rank the bins based on number of segments in each bin or number of base pairs inside segments in each bin. But there are also alternatives like the smallest number of segments or base pairs in the segments in one of the tracks in the suite in the bin in order to require that all the tracks are enriched in the bin.

Hypothesis testing per bin

Question

Are the most enriched bins more enriched than expected by chance?

This may also be formulated as a local question for each of the bin. This would require controlling the false discovery rate, FDR.

Test statistics

We may use the same test statistics as the statistics in the descriptive ranking, i.e. the number of segments or number of base pairs inside segments in the bin. Alternatively, we may use the smallest number of segments or base pairs in segments in one of the tracks in the suite in the bin in order to require that all the tracks are enriched in the bin.

Null model

The segments in each track are distributed independently between the bins. We may define different preservation rules. Preserve the number of segments in the suite of tracks, preserve the segments and intersegments or preserve the bins for each track, but not the order of the bins. Note that the different simulation algorithms define different populations with suites of tracks which may be important to the result of the hypothesis testing since the simulation algorithm defines the interpretation of independent.

Simulation algorithm

We may use different simulation algorithms:

1. The simulation algorithms described previously *track simulation segment-intersegment, suite simulation uniform* or *suite simulation proportional.* We may not use the fixed position simulation algorithms, since we need to move the position of the segments between the bins. As usual, we calculate the test statistic for each simulation to find an estimate of the null distribution.
2. We may use a binomial statistic $O_{i,s} \sim bin(p_{i,s}, n)$ where $O_{i,s}$ is the observed number of segments in bin i and $p_{i,s}$ is the probability for segments to be in bin I, which is proportional with the length of the bin. Alternative definitions of $p_{i,s}$ to have the same value in all bins or use some other information. If the number of bins is large, and the statistic is not dominated by only a few bins, the statistic will be approximately normal.

3. We may assume a multinomial distribution of segments between the bins if we focus on more than one bin in the test statistic. The p-value may be computed either analytically or by distributing the segments uniformly to the bins.
4. We may for each track randomize the ranks of each bin.

The simulation algorithms generate several sets of suites. For each set, we compute the test statistic from a suite, and we find the distributions for this variable. For the binomial and multinomial simulation algorithms, it is possible to calculate the distribution analytically. The distribution is used in the hypothesis tests.

Hypothesis testing on full suite

Question

Are the segments from the tracks more overrepresented in some bins and more underrepresented in other bins than expected by chance?

Test statistics

There are several different alternatives:

1. We may use a kind of chi-square statistic $\chi_{2,s} = \sum_i (O_{i,s} - E_{i,s})^2 / E_{i,s}$, where $O_{i,s}$ is the observed number of segments in bin i and $E_{i,s}$ is the expected number of segments in bin i summed over all tracks in A. The subscript s is used since it is the overlap between number of segments and b is used below when we count base pairs. The expected number $E_{i,s}$ is the total number of segments summed over all tracks in A divided by the number of bins. Alternative definitions of $E_{i,s}$ that include information like for example the length of the bins or a reference track, are also possible. If the number of bins is large, and the statistic is not dominated by only a few bins, the statistic will be approximately normal.
2. We may use the statistic $\chi_{2,b} = \sum_i (O_{i,b} - E_{i,b})^2 / E_{i,b}$ where $O_{i,b}$ is the observed number of base pairs in segments in bin i summed over all tracks in A, and $E_{i,b}$ is the expected number of base pairs in segments in bin i summed over all tracks in A. Here there is a strong correlation between the different base pairs and we will observe more extreme values than for statistic 1 above. The expected number $E_{i,b}$ may be based on the total number of segments in all the tracks and the number of bins. Alternative definitions of $E_{i,s}$ that include information like for example the length of the bins or a reference track, are also possible.
3. For each track we may rank the bins according to decreasing number of segments in the bin. The sum of these ranks for each bin may be used as a test statistic for each bin. The largest of these sums may be used as a test statistic in the global question.
4. We may also use the test statistic $T_8(A_i, A_j, b)$ and take the average value over each pair of tracks. A high value indicates that the tracks in the suite are overrepresented in the same bins.

The chi-square statistic is very strong and will probably give significance for most large datasets. However, the value may be important in comparisons. Note that the position of a segment or point within a bin does not influence the value of any of the three statistics defined above.

Null model and simulation algorithm
The null model and simulation algorithm may be the same as when we have a hypothesis for each bin.

5. In which genomic regions are the tracks of the suite coinciding the most?

Compare a suite of tracks $A = A_1, \dots, A_n$ with segments in a sequence of bins, $b_1, \dots, b_k$.

Descriptive statistics for ranking

Here we want to rank the bins based on the occurrence of the elements of the tracks in the suite. It is possible to argue for several different alternatives in the hypothesis test. A natural choice may be to use $R_{5,3,3}(A_b)$ for each bin as it measures the average interaction with all the other tracks in the suite. Here A_b denotes the suite in each bin b. An alternative is $R_{k,s,2}(A_b)$ for $s = 2$ or $s = 5$ that finds the interaction to the track that has the largest interaction to the remaining tracks. $R_{3,3,3}(A_b)$ or $R_{7,3,3}(A_b)$ may also be a natural choices.

Hypothesis testing per bin

Question
Do the segments coincide more than expected in the bin where the segments coincide most?

Test statistics
We may use the same test statistics as the statistics in the descriptive ranking.

Null model and simulation algorithm
The null model may be the same as for question 4 on overrepresentation. The simulation algorithm may be *simulation segment-intersegment, suite simulation uniform* or *suite simulation proportional*. We may not use the fixed position simulation algorithms, since we need to move the position of the segments between the bins. As usual, we calculate the test statistic for each simulation to find an estimate of the null distribution.

Hypothesis testing on full suite

Question
Do the segments coincide more than expected in some bins and less than expected in other bins?

Test statistic
Here we want to find out whether the segments coincide more in some bins and less in other bins than expected. Hence that the variability of $R_{k,s,2}(A_b)$ is larger than expected. Then we may use a statistics like

$$\chi_{2,R} = \sum_b (R_{k,s,2}(A_b) - \frac{1}{\#b}\sum_b R_{k,s,2}(A_b))^2 \,/(\frac{1}{\#b}\sum_b R_{k,s,2}(A_b))$$

where $\#b$ is the number of bins.

Null model and simulation algorithm
The null model and simulation algorithm may be the same as for the hypothesis test for each bin.

Correspondence between terminology in the web interface and this supplementary note

In the menu of the web interface, we provide short form names for tools corresponding to questions of this supplementary note (after selecting a tool, a full name is displayed in the header, corresponding to what is used in this note). The mapping between questions and short form names in the menu is as follows:

- Which tracks (in a suite) are most representative and most atypical?
  -> Determine representative and atypical tracks in a GSuite
- Which tracks (in a suite) coincide most strongly with a separate single track?
  -> Determine GSuite tracks coinciding with a target track
- Are certain tracks of one suite coinciding particularly strongly with certain tracks of another suite?
  -> Determine coinciding track combinations from two suites
- Which genomic regions are the most enriched with points/segments of the tracks of the suite?-> Determine regions where GSuite tracks are enriched
- In which genomic regions are the tracks of the suite coinciding the most?-> Determine regions where GSuite tracks co-occur more strongly

For choosing descriptive and test statistics in the web interface, we provide textual descriptions of each statistic. In this note, we prefer a short form of indexes for a variable T. The mapping between textual descriptions in the interface and T-indices are as follows:

- Forbes coefficient: ratio of observed to expected overlap
  -> T5
- Normalized Forbes coefficient: ratio of observed to expected overlap normalized in relation to the reference GSuite -> T7
- Jaccard index: ratio of overlapping base-pairs relative to the union base-pairs -> T3
- Proportion of the query track base-pairs coinciding with base-pairs from the reference track -> T1
- Proportion of the union of base-pairs of the two tracks that are covered by the reference track -> T2
- Ratio of probability in being inside the intersection and the geometric mean of being inside each track -> T4
- Correlated bin coverage -> T8

Similarly, where the web interface provides descriptions of alternative ways of summarizing T statistics, we in the note preferred indexed variations of a variable Q. To represent the different Qs, the web interface uses the combination of two input fields:

- A summary function, one of {average, minimum, maximum} is selectable
- Reversed, when checked the tracks $T_k(A_i,\ A_j)$ is replaced by $T_k(A_j,\ A_i)$ in the calculation.

The mapping to the Qs is then straightforward.

Finally, for analyses in the web interface based on suite-level statistics (corresponding to R-statistics in this note), the summarization of Q-values is always set to take the average (i.e. the last index of R-statistics are consistently set to 3 for analyses through the web interface).

Appendix

Here we describe some more detailed calculations.

We assume the statistical properties of the two segment tracks A_i and A_j are fixed and we want to find the statistical properties of the overlap. It is necessary to make some approximations in order to find an expression for the overlap. Here we will assume that the statistical properties of each of the tracks are fixed, $bp(A_i)/N, bp(A_i)/N \ll 1$ and that the length distribution of the segments is the same in the two tracks. We may write that the overlap $O = bp(A_i \cap A_j)/N = XZ$, where X and Z are two independent events, the number of overlaps divided by N, X, and the length of an overlap, Z. We have approximately that $X \sim bin(bp(A_i)/E(L), 2bp(A_j)/N)$ where $E(L)$ is the average length of a segment. The expression $bp(A_i)/E(L)$ is the number of segments in A_i and $2bp(A_j)/N$ is the probability that it overlaps with a segment in A_j. If the track A_i had been a point track, the probability would have been $bp(A_j)/N$. Since the first point in a segment in A_i may be outside the segments in A_j and the average length of segments, $E(L)$, is the same in the two tracks, the probability for an overlap is doubled. Then $Var(X) \approx E(X)$=$2bp(A_i)bp(A_j)/(N^3E(L)) \ll 1$.

We use the general formula that $Var(XZ) = Var(X)(\frac{Var(Z)}{2} + (E(Z))^2) + Var(Z)(E(X))^2$. In our case, we may neglect the term $Var(Z)(E(X))^2$.

Then we have $Var(O) \approx \ bp(A_i)bp(A_j)(Var(Z) + 2E(Z)^2)/(N^3E(L))$.

Let L be the length of a segment. We have $E(Z) \approx E(L)^2/E(L)$. If one of the tracks was a point track, then the formula would have been exact. As a very rough approximation, we may also set $Var(Z) \approx E(L)^2$. If the length distribution of segments in the tracks varies much, it may be necessary with better approximation of the expectation and variance of Z. This may require simulation.

This makes it possible to find $Var(\ T_5(A_i, A_j)) \approx \ N(Var(Z) + 2E(Z)^2)/(bp(A_i)bp(A_j)E(L))$.

References

[1] Jaccard, Paul (1912), The distribution of the flora in the alpine zone, New Phytologist 11: 37–50, doi:10.1111/j.1469-8137.1912.tb05611.x

[2] Hayek, L.-A. C. 1994. Analysis of amphibian biodiversity data. Pp 207-269. In Measuring and monitoring biological diversity. Standard Methods for Amphibians. Eds., W.R. Heyer et al. Smithsonian Institution, Washington, D.C.

[3] Sandve, Geir Kjetil; Gundersen, Sveinung; Rydbeck, Halfdan; Glad, Ingrid Kristine; Holden, Lars; Holden, Marit; Liestøl, Knut; Clancy, Trevor; Drabløs, Finn; Ferkingstad, Egil; Johansen, Morten;

Nygaard, Vegard; Tøstesen, Eivind; Frigessi, Arnoldo; Hovig, Eivind. The differential disease regulome. BMC Genomics (ISSN 1471-2164). 12 pp 353. doi: 10.1186/1471-2164-12-353. 2011.
