# Supplementary material for: GSuite HyperBrowser: integrative analysis of dataset collections across the genome and epigenome
Source: Gigascience. 2017 Apr 27;6(7):1–12. doi: 10.1093/gigascience/gix032 (PMC5493745; doi:10.1093/gigascience/gix032)
Supplement: Additional File 2: — A text document providing a critical evaluation (on simulated and real data) of how particular choices of similarity measures may influence genome-level analysis results (PDF format, 1233 KB). [file gix032_Additional_file_2.pdf]

Additional file 2: An exploration of similarity measures

Simovski et al., "GSuite HyperBrowser: integrative analysis of dataset collections across the genome and epigenome"

1 Genomic features co-occurring with GATA1

1.1 GATA1 biology

GATA1 is an appropriate transcription factor (TF) to focus on in the K562 cell line, a chronic myelogenous leukemia cell line, since GATA1 is the TF with the highest expression in these cells according to Fantom5 (http://fantom.gsc.riken.jp/5/sstar/FF:10454-106G4, http://fantom.gsc.riken.jp/5/sstar/FF:10824-111C5). GATA1 is a zinc-finger type TF with a key role in regulating gene programs during haematopoiesis, where it induces megakaryocytic and erythroid commitment and simultaneously prevents granulocyte-monocyte and lymphoid development [1, 2, 3]. GATA1 is the founding member of a family of related TFs where the three most studied members, GATA1, GATA2 and GATA3, play unique and essential roles in the development of particular haematopoietic cell lineages [3, 4, 5]. Here, GATA1 is essential for erythroid commitment and differentiation through the activation of a variety of genes that is required for red blood cell formation [2]. GATA1 is a sequence-specific DNA-binding protein recognizing recognising the GATA box motif (WGATAR) found in regulatory sites controlling its target genes [6]. Approximately 20,000 GATA1 bound sites are found in the genome of erythroid cells [7]. GATA1 binding may lead to both repression and activation depending on context, the latter probably being determined by the combinatorial pattern of TFs bound at the regulatory region(s) [8, 9, 10, 11]. GATA1 exerts its role as a master regulator of erythropoiesis by operating in DNA-associated multimeric complexes including other TFs such as TAL1, KLF1, and NFE2 as well as the nonDNA-binding TFs such as LMO2 and LDB1 [12, 7, 13, 14, 15, 16]. A recent study, which used high-resolution ChIP-exo to map GATA1 and TAL1 across the mouse genome, identified ~10,000 GATA1 and ~15,000 TAL1 bound sites, of which ~4,000 locations were bound by both GATA1 and TAL1 [17]. The current understanding is that key complexes are formed by combinatorial occupancy patterns of erythroid TFs leading to the induction of key erythroid genes.

1.2 Identified co-occupancy partners (Forbes)

To explore co-occupancy with GATA1 in K562 cells, we collected 318 protein and histone modification ChIP-seq peak tracks from ENCODE [18] and downloaded to history. We then performed similarity testing of one GATA1 ChIP-seq peak track (Snyder lab, Stanford) against the 317 peak tracks using the Forbes similarity measure [19]. Figure 1 shows the top 20 ranking list of protein peak tracks similar

to GATA1 as they appeared in the results. Evaluating the full ranking list for co-occupancy with GATA1 (see History element 32), there are six peak tracks amongst the top 20 for GATA1 and GATA2, with another separately generated ChIP-seq peak track for GATA1 ranked first. As described above, GATA1 and GATA2 recognise similar GATA box motifs due to high conservation in their zinc-finger like domains [20], and we would therefore anticipate that GATA2 can show high co-occurrence with GATA1. One exception is a track EGFP-GATA2 (ranked position 70), which at closer inspection may be due to the use of a different peak-calling algorithm on data from the same experiment as used for the track ranked at position 8.

Rank	antibody	Track title	Similarity to query track
1	GATA-1	wgEncodeSydhTfbsK562Gata1UcdPk.narrowPeak	211.770367075
2	Brg1	wgEncodeSydhTfbsK562Brg1IggmusPk.narrowPeak	187.655025077
3	SIRT6	wgEncodeSydhTfbsK562Sirt6StdPk.narrowPeak	184.706529972
4	GATA-1	wgEncodeAwgTfbsSydhK562Gata1UcdUniPk.narrowPeak	178.274481689
5	GATA-2	wgEncodeSydhTfbsK562Gata2UcdPk.narrowPeak	169.259335782
6	SIRT6	wgEncodeAwgTfbsSydhK562Sirt6UniPk.narrowPeak	160.518868713
7	Ini1	wgEncodeSydhTfbsK562Ini1IggmusPk.narrowPeak	131.350025311
8	eGFP-GATA2	wgEncodeAwgTfbsUchicagoK562Egata2UniPk.narrowPeak	127.228660564
9	GATA-2	wgEncodeAwgTfbsSydhK562Gata2UcdUniPk.narrowPeak	122.057567691
10	Brg1	wgEncodeAwgTfbsSydhK562Brg1IggmusUniPk.narrowPeak	119.604152022
11	STAT5A_(SC-74442)	wgEncodeAwgTfbsHaibK562Stat5asc74442V0422111UniPk.narrowPeak	118.209993778
12	TBLR1_(NB600-270)	wgEncodeAwgTfbsSydhK562Tblr1nb600270IggrabUniPk.narrowPeak	113.917745686
13	ZNF274_(M01)	wgEncodeAwgTfbsSydhK562Znf274m01UcdUniPk.narrowPeak	112.038365683
14	STAT2	wgEncodeAwgTfbsSydhK562Stat2Ifna30UniPk.narrowPeak	106.887602348
15	BCL3	wgEncodeAwgTfbsHaibK562Bcl3Pcr1xUniPk.narrowPeak	102.787263899
16	COREST_(ab24166)	wgEncodeAwgTfbsSydhK562Corestab24166IggrabUniPk.narrowPeak	101.133113219
17	GATA2_(SC-267)	wgEncodeAwgTfbsHaibK562Gata2sc267Pcr1xUniPk.narrowPeak	98.8594841276
18	STAT1	wgEncodeAwgTfbsSydhK562Stat1Ifna30UniPk.narrowPeak	95.6667172657
19	STAT2	wgEncodeAwgTfbsSydhK562Stat2Ifna6hUniPk.narrowPeak	88.7855960513
20	Ini1	wgEncodeAwgTfbsSydhK562Ini1IggmusUniPk.narrowPeak	88.4991080644

Figure 1 Ranked list of proteins similar to GATA1 according to the Forbes similarity measure

Brahma related gene 1 (BRG1/SMARCA4) was ranked as a highly similar dataset to GATA1 (ranked position 2 and 10) using the Forbes similarity measure. BRG1 is an ATPase component of the SWI/SNF chromatin remodelling complex that has been shown to co-localize and interact with TAL1 and GATA1 [21, 22]. Moreover, a study from Zhao and colleagues has shown that the remodelling activity of BRG1 shifts nucleosomes to open up GATA1 binding sites during haematopoietic stem cell differentiation to the erythrocyte lineage [23]. Their data suggests that GATA1 recruits BRG1 and facilitates binding of TAL1 at enhancers. We were therefore expecting that TAL1, which is found to be in a multimeric complex with GATA1, would show a co-occurrence with the GATA1. However, the peak track for TAL1 was ranked at position 31 and 43 (same ChIP experiment, two different peak tracks) using the Forbes similarity measure. Another co-factor we have discussed above

to be implicated in a complex with GATA1, NFE2, had an even lower ranking using Forbes similarity measure (position 144 and 145, same ChIP experiment, two different peak tracks). An integrative analysis described more in detail below has identified a cluster of GATA1, GATA2 and c-Jun with SIRT6, BRG1 and INI1 [24]. Using Forbes similarity measure, GATA1, GATA2, SIRT6, INI1, BRG1 and c-Jun peak tracks are all ranked at the top. The 20 datasets that showed least co-occupancy with GATA1 using Forbes similarity measure can be divided in four categories: chromatin sites of boundaries and looping (insulator proteins CTCF and cohesin complex component Rad21 [25], RNA polymerase II (RNAPII) transcription cycles (pausing factor NELF (negative elongation factor) and active gene body elongation Pol2S2P and H3K36me3) [26], repressed chromatin (H3K27me3 [27], ZNF274 and SETDB1/ESET [28, 29], RNAPIII transcription (BRF1 and Pol3 [30]). GATA1 occupies promoters and enhancers, and we therefore expect less overlap with RNAPII elongation and H3K36me3 and this fits with the ranking using Forbes similarity measure. That all the CTCF peak tracks show little similarity with GATA1 peak tracks using the Forbes measure is surprising since GATA1 has been implicated in chromatin looping of the globin locus [31, 32] via its interaction with BRG1 [33] and found to colocalize with GATA2 and CTCF at Kaiso binding sites in K562 cells [34]. GATA1 recruitment of TAL1 is also required for chromatin looping [35]. However, as described above, TAL1 peak tracks were not at the top ranking using Forbes similarity measure. The H3K9 methyltransferase SETDB1 was not found to co-occur with GATA1 using Forbes similarity measure, however peak tracks for TRIM28/KAP1 and ZNF274 that interact in complex with SETDB1 [28, 36], were found both at the top (position 13/40 and 23) and bottom of the ranking list, which shows that there is some biological inconsistencies in this analysis. Generally, histone mark ChIP-seq peak tracks have broader genome coverage than DNA binding factors, and we included histone peak tracks for H3K4me1, H3K4me3, H3K9ac, H3K27me3, and H3K36me3 in our analysis. All these histone mark peak tracks had a lower ranking when compared to occupancy of GATA1 (the first histone peak track was the repressive mark H3K27me3 at position 120) using the Forbes similarity measure.

1.3 Identified co-occupancy partners (Jaccard)

We then explored similarity of the GATA1 ChIP-seq peak track (Snyder lab, Stanford) against the 317 experimental datasets using the Jaccard similarity measure [37]. Figure 2 shows the top 20 ranked list of proteins similar to GATA1 as they appeared in the results. Evaluating the full list for co-occupancy with GATA1 (see History element 34), five GATA2 tracks were ranked amongst the top 20. Contrary to Forbes similarity measure, which ranked several GATA1 tracks highly similar, the first GATA1 peak track is ranked at position 42 with Jaccard similarity measure. However, the GATA1 partner TAL1, which was not ranked highly similar to GATA1 in the Forbes list above, was ranked at positions 3 and 11 (same ChIP experiment, two different peak tracks) using the Jaccard similarity measure (Figure 2). Furthermore, the histone acetyltransferase p300 has no DNA binding domain, but is known to interact with many TFs, and its close relative CBP has been identified to be a co-factor of GATA1 [38, 39]). Using Jaccard similarity measure, p300 was ranked in the list at position 5, 6 and 23, but also at position 194. The ARID family has previously

been linked to SWI/SNF chromatin remodelling as an alternative component to BRG1 [40], and ARID3A was listed at position 12 using the Jaccard similarity measure. BRG1, ranked very similar to GATA1 using Forbes, was only ranked at position 60 and 95 using Jaccard similarity measure. STAT5A and TRIM28 were identified as top hits using both Forbes and Jaccard similarity measures. All the histone mark datasets (H3K4me1, H3K4me3, H3K9ac, H3K27me3, and H3K36me3) had also a lower ranking when compared to occupancy of GATA1 using the Jaccard similarity measure. The most highly ranked histone datasets were for enhancer mark H3K4me1 at position 87 and 88, which has a wide genome coverage. Amongst the lowest Jaccard ranking peak tracks, several tracks were also found least similar to GATA1 using Forbes. These were Pol2S2P, NELF, Pol3, BRF1 and 2, H3K27me3 and ZNF274.

Rank	antibody	Track title	Similarity to query track
1	eGFP-GATA2	wgEncodeAwgTfbsUchicagoK562Egata2UniPk.narrowPeak	0.185890040051
2	GATA-2	wgEncodeSydhTfbsK562Gata2UcdPk.narrowPeak	0.152994212293
3	TAL1_(SC-12984)	wgEncodeSydhTfbsK562Tal1sc12984IggmusPk.narrowPeak	0.149817362079
4	eGFP-GATA2	wgEncodeUchicagoTfbsK562Egata2ControlPk.narrowPeak	0.132033963091
5	p300	wgEncodeSydhTfbsK562P300IggrabPk.narrowPeak	0.125599260943
6	p300	wgEncodeAwgTfbsSydhK562P300IggrabUniPk.narrowPeak	0.119147165537
7	GATA2_(SC-267)	wgEncodeAwgTfbsHaibK562Gata2sc267Pcr1xUniPk.narrowPeak	0.114637700254
8	PML_(SC-71910)	wgEncodeAwgTfbsHaibK562Pmlsc71910V0422111UniPk.narrowPeak	0.113955674094
9	TBLR1_(NB600-270)	wgEncodeSydhTfbsK562Tblr1nb600270IggrabPk.narrowPeak	0.113417860712
10	TEAD4_(SC-101184)	wgEncodeAwgTfbsHaibK562Tead4sc101184V0422111UniPk.narrowPeak	0.112068986772
11	TAL1_(SC-12984)	wgEncodeAwgTfbsSydhK562Tal1sc12984IggmusUniPk.narrowPeak	0.111221320688
12	ARID3A_(sc-8821)	wgEncodeSydhTfbsK562Arid3asc8821IggrabPk.narrowPeak	0.111088642708
13	GATA-2	wgEncodeAwgTfbsSydhK562Gata2UcdUniPk.narrowPeak	0.109121287804
14	STAT5A_(SC-74442)	wgEncodeAwgTfbsHaibK562Stat5asc74442V0422111UniPk.narrowPeak	0.108210689621
15	TRIM28_(SC-81411)	wgEncodeAwgTfbsHaibK562Trim28sc81411V0422111UniPk.narrowPeak	0.103995326471
16	COREST_(ab24166)	wgEncodeSydhTfbsK562Corestab24166IggrabPk.narrowPeak	0.102135723281
17	CCNT2	wgEncodeSydhTfbsK562Ccnt2StdPk.narrowPeak	0.101017116549
18	ZNF-MIZD-CP1_(ab65767)	wgEncodeSydhTfbsK562Znfmizdcp1ab65767IggrabPk.narrowPeak	0.100329120986
19	CDP_(sc-6327)	wgEncodeSydhTfbsK562Cdpsc6327IggrabPk.narrowPeak	0.100216195111
20	ATF1_(06-325)	wgEncodeSydhTfbsK562Atf106325StdPk.narrowPeak	0.100052452293

Figure 2 Ranked list of proteins similar to GATA1 according to the Jaccard similarity measure

1.4 Identified co-occupancy partners using tetrachoric correlations, including a comparison with the Jaccard and Forbes similarity measures

We next explored similarity of the GATA1 ChIP-seq peak track (Snyder lab, Stanford) against the 317 experimental peak tracks using a third similarity measure, the tetrachoric correlation [41, 42, 43] (see Section 2 for a definition and further discussion). Figure 3 shows the resulting ranked list of top 20 proteins similar to GATA1. In the full ranking list (see History element 15) the enrichment of GATA factors at the top of the ranked list is more pronounced than in the other two lists

(Forbes and Jaccard similarity measures). The five highest ranked positions are in fact occupied with GATA1 and GATA2 tracks, with two additional GATA2 tracks at position 9 and 13. Thus six of the top ten (or 7 of the top 20) are GATA tracks. This is a somewhat stronger enrichment at the top than found with the two other similarity measures, where Forbes ranked six and Jaccard five GATA tracks among the top twenty lists. Using tetrachoric correlation, the three highest ranked TFs apart from GATA itself were TAL1, BRG1 and p300, which are all of high biological relevance as discussed above. The next on the list, STAT5A seems relevant, given reports that STAT5, like GATA1, is associated with induced erythropoiesis [44], and STAT5-induced erythropoiesis is GATA1-dependent [45]. Using the Forbes similarity measure, GATA1 showed high similarity with GATA2, c-Jun, SIRT6, BRG1 and INI1 [24]. However, using the tetrachoric correlation only SIRT6 and BRG1 have top ranking (position 12,15 and 6,21, respectively), while c-Jun and INI1 were ranked lower on the list (position 40 and 31 respectively). NFE2, which is a well known GATA1 interacting protein, had low ranking in the lists of the tetrachoric correlation as well as Forbes and Jaccard similarity measures.

Rank	antibody	Track title	Similarity to query track
1	eGFP-GATA2	wgEncodeUchicagoTfbsK562Egata2ControlPk.narrowPeak	0.83758869959
2	GATA-2	wgEncodeSydhTfbsK562Gata2UcdPk.narrowPeak	0.830204595413
3	eGFP-GATA2	wgEncodeAwgTfbsUchicagoK562Egata2UniPk.narrowPeak	0.815536656054
4	GATA-1	wgEncodeSydhTfbsK562Gata1UcdPk.narrowPeak	0.807826090189
5	GATA-1	wgEncodeAwgTfbsSydhK562Gata1UcdUniPk.narrowPeak	0.787937209944
6	Brg1	wgEncodeSydhTfbsK562Brg1IggmusPk.narrowPeak	0.774430088134
7	TAL1_(SC-12984)	wgEncodeSydhTfbsK562Tal1sc12984IggmusPk.narrowPeak	0.768866980484
8	p300	wgEncodeSydhTfbsK562P300IggrabPk.narrowPeak	0.758131642204
9	GATA-2	wgEncodeAwgTfbsSydhK562Gata2UcdUniPk.narrowPeak	0.757527113318
10	STAT5A_(SC-74442)	wgEncodeAwgTfbsHaibK562Stat5asc74442V0422111UniPk.narrowPeak	0.752568551431
11	eGFP-JunD	wgEncodeUchicagoTfbsK562EjundControlPk.narrowPeak	0.742176694274
12	SIRT6	wgEncodeAwgTfbsSydhK562Sirt6UniPk.narrowPeak	0.739864813573
13	GATA2_(SC-267)	wgEncodeAwgTfbsHaibK562Gata2sc267Pcr1xUniPk.narrowPeak	0.736159536553
14	TBLR1_(NB600-270)	wgEncodeAwgTfbsSydhK562Tblr1nb600270IggrabUniPk.narrowPeak	0.73519568689
15	SIRT6	wgEncodeSydhTfbsK562Sirt6StdPk.narrowPeak	0.730199652218
16	COREST_(sc-30189)	wgEncodeSydhTfbsK562Corestsc30189IggrabPk.narrowPeak	0.717901614208
17	TBLR1_(NB600-270)	wgEncodeSydhTfbsK562Tblr1nb600270IggrabPk.narrowPeak	0.716919293429
18	p300	wgEncodeAwgTfbsSydhK562P300IggrabUniPk.narrowPeak	0.712914036123
19	TRIM28_(SC-81411)	wgEncodeAwgTfbsHaibK562Trim28sc81411V0422111UniPk.narrowPeak	0.710655393535
20	PML_(SC-71910)	wgEncodeAwgTfbsHaibK562Pmlsc71910V0422111UniPk.narrowPeak	0.707856785972

Figure 3 Ranked list of proteins similar to GATA1 according to the Tetrachoric Correlation

As described above, the 23 histone peak tracks had a low ranking position for both Forbes and Jaccard measures. Similar to the Jaccard measure, the most highly ranked histone peak track was the H3K4me1 enhancer mark, having ranking positions 75 and 76 using the tetrachoric correlation measure. Moreover, tetrachoric correlations was similar to Forbes for low co-occupancy of CTCF with GATA1 (grouped ranking

positions 276, 279, 285-88 and 290-92). Amongst the lowest ranking peak tracks in the tetrachoric correlation list, several datasets were also found least similar to GATA1 using Forbes and Jaccard similarity measures. These were Pol2S2P, NELF, Pol3, BRF1 and 2, H3K27me3 and ZNF274.

1.5 Conclusions drawn from three similarity measures with GATA1

We find that the tetrachoric correlation, Forbes and Jaccard similarity measures have strengths and weaknesses judged from a biological point of view. The GATA1 co-occupancy partner list according to Forbes contains several more specific proteins including chromatin-modulating factors, such as BRG1 and SIRT6, and TFs such as other GATA1 peak tracks, GATA2 and STAT5A, which appear as an advantage because the list may generate specific questions that can be addressed experimentally. The corresponding top ranked GATA1 partners according to Jaccard similarity measure are associated with other GATA-family members binding the same sites and some general factors such as p300, which are associated with most transcriptional processes. There is however one apparently strong point of the Jaccard list, and that is the high ranking of TAL1. TAL1 is ranked on place 3 and 11 in the Jaccard list, while only being ranked on place 31 and 43 in the Forbes list. Also the Tetrachoric Correlation list has TAL1 quite high, at place 7 and 22. Many reports have found GATA1 and TAL1 to be jointly responsible for proper erythroid gene expression [46, 47, 48].

If we compare the top-twenty in the tetrachoric correlation versus Forbes and Jaccard lists of similarity measures for GATA1, we notice that the number of TFs with known biological relevance (GATA1, GATA2, TAL1, STAT5A) is seven for Forbes, eight for Jaccard and nine for Tetrachoric Correlation. If we count the number of typical epigenetic modifiers in the same top-twenty (p300, BRG1, SIRT6), we obtain four for Forbes, two from Jaccard and five from Tetrachoric Correlation. The latter therefore seems to outperform the two others in selecting both specific DNA-binding factors and their cooperating chromatin modifiers.

The current analysis is performed ab initio without any filtering based on prior knowledge of GATA1 biology. It was of great interest to find what such a direct procedure can reveal about GATA1-biology. In addition to some reasonable associations discussed above, there are also limitations, in the sense of variable ability to identify co-occupancy partners expected from established knowledge of GATA1. This concerns in particular the TF TAL1, but also NFE2 (the other cofactors KLF1, LMO2 and LDB1 are not represented in the list of 317 peak tracks). It is interesting to note that such well-known co-occupancy partners are not necessarily apparent through a direct co-occurrence approach as taken here. More sophisticated analyses using measures tailored to capture TF co-occupancy might be able to detect the expected TF relations, but is beyond the scope of this investigation. The ENCODE data for GATA1 has previously been analysed by Ulirsch et al. [7] where they addressed the conservation of gene regulatory networks between the human and mouse system, exemplified by GATA1. Several interesting conclusions were drawn. While 75% of the GATA1 bound sites were not conserved between species, those that were conserved were enriched in cis-regulatory modules (CRMs) co-occupied by the three master regulators GATA1, KLF1, and TAL1. Such co-occupied CRMs

are under strong evolutionary constraint and localize to genes that are part of gene regulatory programs defining the erythroid cell state. A similar principle for many TFs was confirmed by an extended analysis reported by Cheng et al. [49] where they concluded that occupancy-conserved TF-bound sequences tend to function in several tissues and also co-associate with many TFs. Hence, one limitation in the current analysis is that there is no filtering and all GATA1 peaks are judged as equally important for the processes regulated by GATA1. If 75% of these peaks are not conserved, this group is probably not as essential as the conserved ones. And if conservation is linked to co-occupancy of several regulators, this means that key biological functions may be associated with only a subset of occupied sites. GATA1 has been mostly studied because of its role in defining cell fate during erythropoiesis. The recent unraveling of super-enhancers has shown that cell fate often is linked to these large regions of enhancers where several regulators cluster [50, 51]. Because super-enhancers represent only a small fraction of the total number of enhancers [50], it seems that an unfiltered approach as in the present ranking of all peaks, may not reveal some key regulatory networks linked to cell fate determination and differentiation, which are so tightly associated with GATA1-biology. Instead, this analysis will provide more global preferred partners and for this we suggest the tetrachoric correlation analysis to be the preferred similarity measure. A last point to this analysis is that the quality of the ChIP experiments, including the genome-coverage due to choice of antibody, chromatin shearing and sequencing depth and the peak-calling algorithm, may influence the final ranking results. Therefore, a quality threshold of datasets may improve the analysis.

2 Influence of track size on the tetrachoric correlation, Forbes and Jaccard similarity measures

We here provide an exploration of track size-related biases of the tetrachoric correlation, Forbes and Jaccard similarity measures, using the question of "which tracks (in a suite) coincide most strongly with a separate single track" as an example. These considerations apply similarly to statistics related to other statistical questions considered in the GSuite HyperBrowser manuscript.

For our selected question, we want to rank a set of reference tracks R_i based on how much they co-occur with a query track Q, by measuring the similarity of every reference track to the query track, and rank the reference tracks based on this measure. We discuss aspects that should be taken into account when choosing a suitable statistic for this purpose. We define a track $S \subset G$ as a subset of genome positions $G = 1, 2, ..., N$ and the complement $S^C = b \in G : b \notin S$.

We start by defining the three different similiarity measures. Frist, the tetrachoric similiary measure [41, 42, 43] is defined by assuming that the two tracks R_i and Q are generated by thresholding an underlying continuous, bivariate normally distributed variable, and the tetrachoric correlation ρ is then defined as the correlation in the underlying bivariate normal. In general, the thresholds will be different for R_i and Q, and both the thresholds and the correlation ρ can be easily estimated from given tracks, for example using maximum likelihood techniques. We have used the R-package polycor [52] to estimate ρ. Second, the Forbes similarity measure is simply defined as $\frac{N|R_i \cap Q|}{|R_i||Q|}$, where we use the notation $|A|$ for the number of elements of a set A. Finally, the Jaccard index is defined as $\frac{|R_i \cap Q|}{|R_i \cup Q|}$.

2.1 Demonstration of track size influence using simulated data

Ideally, a similarity measure should not be affected by track size, since the track size in itself is not relevant for the question of whether two tracks are similar/related. The Jaccard similarity measure has been seen to be strongly affected by track size, even in the case of independence between tracks, while the tetrachoric and Forbes measures are unaffected under independence. We will now demonstrate this on simulated data. Figure 4 shows the three similarity measures calculated for independent tracks of different sizes. In the simulations, both tracks were constructed independently as a set of k randomly selected positions in the range $0..N$ (k randomly selected positions of a genome of size N). N was set to 10^9 in all simulations. For each measure and for a set of discrete values k, $M = 2000$ independent instances were simulated. The solid lines shows the mean simulated values for each measure (y-axis) as a function of k/N (x-axis), while the dashed lines show the mean plus/minus three standard deviations. It is obvious from Figure 4 that the Jaccard measure is strongly affected by track size, while the two other measures are unaffected under independence.

2.2 A quantitative analysis of the tetrachoric, Jaccard and Forbes measures on real data: The effect of subsampling tracks

In this section, we study the result of subsampling on the same set of real biological tracks as discussed in Section 1. A set of 1000 elements were randomly drawn from each of the tracks. Figure 5 shows the results for the Forbes, Jaccard and tetrachoric correlation similarity measures, respectively. Clearly, the Jaccard measure becomes much lower when the tracks are subsampled, while the Forbes and tetrachoric measures do not seem to affected in any significant way.

3 Availability

All results discussed here can be inspected and reproduced by accessing Galaxy histories of the performed analysis at the following URLs.

Ranking of experimental datasets based on similarity to GATA1 according to three different measures:

https://hyperbrowser.uio.no/hb/u/sandve/h/gata1-example---the-effect-of-similarity-measure

Analysis of stability of measures after subsampling:

https://hyperbrowser.uio.no/hb/u/sandve/h/gata1-example---effects-of-subsampling

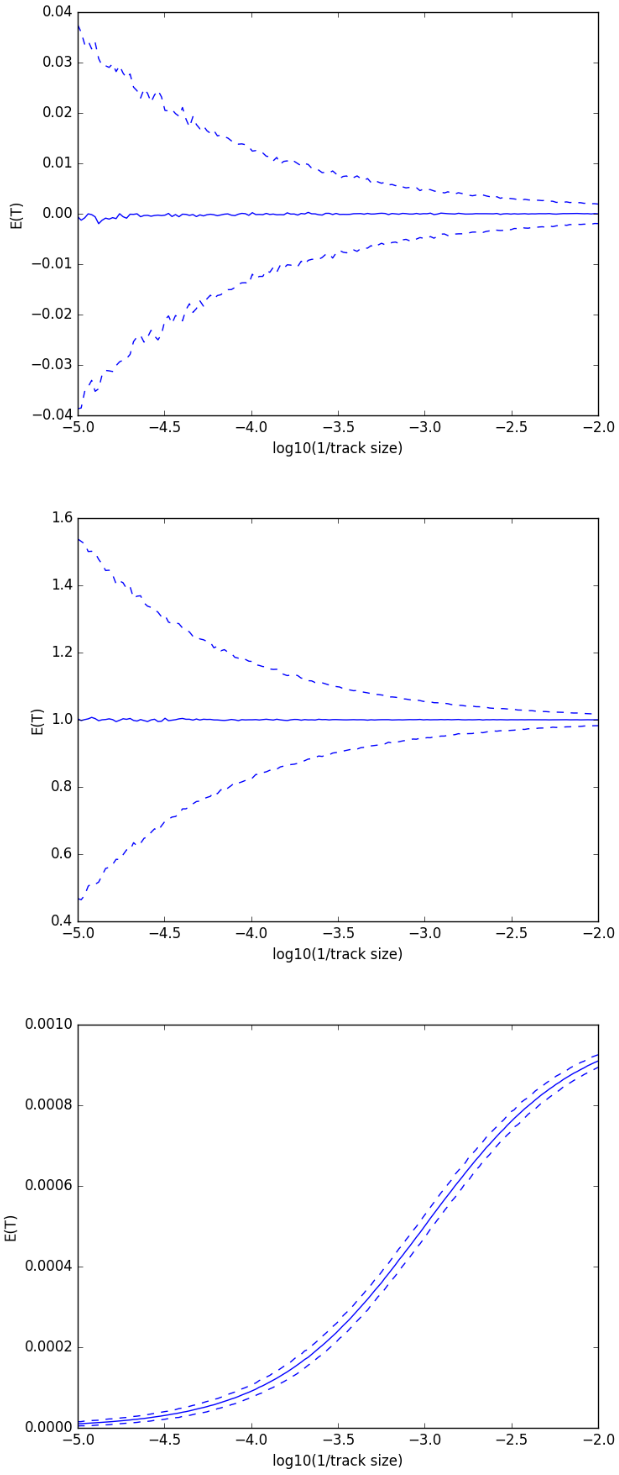

Figure 4 Effect of track size on similiarity measures. The top panel shows results for the tetracoric correlation measure, the middle panel shows results for the Forbes measure, while the bottom panel shows results for Jaccard measure.

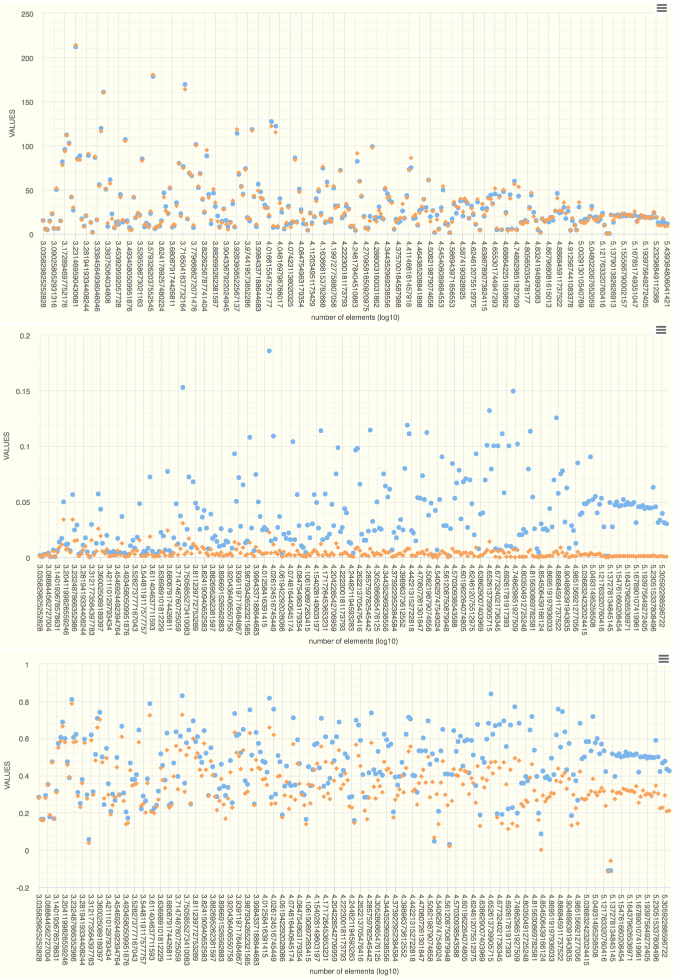

Figure 5 Effect of subsampling on similarity according to the three different considered measures. The logarithmic x-axis gives the number of elements in a given track originally (all tracks have 1000 elements after subsampling), ranging from 1000 to 200 000 (log10-values from 3 to5.3). For each of the three panels, the linear y-axis gives the similarity of tracks before subsampling in blue, and after subsampling in orange. In other words, there will for each track be two points (one blue, one orange) representing the measured similarity for the same track before and after subsampling, located at the exact same x-value since x represents the number of elements in the track before subsampling. With Forbes similarity (top panel), the measured similarities are roughly the same before and after subsampling. As the Jaccard measure (middle panel) is strongly affected by track size, the measured similarities are consistently very low after subsampling (orange points have consistently low y-values). With tetrachoric correlation, the measured correlations are systematically lower after subsampling, but much less pronounced than for Jaccard. The effect is most pronounced for tracks that originally had many elements (where subsampling was strongest).

Author details

References

1. Kitajima, K., Zheng, J., Yen, H., Sugiyama, D., Nakano, T.: Multipotential differentiation ability of GATA-1-null erythroid-committed cells. Genes & Development **20**(6), 654--659 (2006). doi:10.1101/gad.1378206. http://genesdev.cshlp.org/content/20/6/654.full.pdf+html
2. Welch, J.J., Watts, J.A., Vakoc, C.R., Yao, Y., Wang, H., Hardison, R.C., Blobel, G.A., Chodosh, L.A., Weiss, M.J.: Global regulation of erythroid gene expression by transcription factor GATA-1. Blood **104**(10), 3136--3147 (2004). doi:10.1182/blood-2004-04-1603. http://www.bloodjournal.org/content/104/10/3136.full.pdf
3. Ferreira, R., Ohneda, K., Yamamoto, M., Philipsen, S.: GATA1 function, a paradigm for transcription factors in hematopoiesis. Mol Cell Biol **25**(4), 1215--27 (2005). doi:10.1128/MCB.25.4.1215-1227.2005
4. Lentjes, M.H.F.M., Niessen, H.E.C., Akiyama, Y., de Bruïne, A.P., Melotte, V., van Engeland, M.: The emerging role of GATA transcription factors in development and disease. Expert Rev Mol Med **18**, 3 (2016). doi:10.1017/erm.2016.2
5. Gao, J., Chen, Y.-H., Peterson, L.C.: GATA family transcriptional factors: emerging suspects in hematologic disorders. Exp Hematol Oncol **4**, 28 (2015). doi:10.1186/s40164-015-0024-z
6. Trainor, C.D., Omichinski, J.G., Vandergon, T.L., Gronenborn, A.M., Clore, G.M., Felsenfeld, G.: A palindromic regulatory site within vertebrate GATA-1 promoters requires both zinc fingers of the GATA-1 DNA-binding domain for high-affinity interaction. Molecular and Cellular Biology **16**(5), 2238--2247 (1996)
7. Ulirsch, J.C., Lacy, J.N., An, X., Mohandas, N., Mikkelsen, T.S., Sankaran, V.G.: Altered chromatin occupancy of master regulators underlies evolutionary divergence in the transcriptional landscape of erythroid differentiation. PLoS Genet **10**(12), 1--19 (2014). doi:10.1371/journal.pgen.1004890
8. Grass, J.A., Boyer, M.E., Pal, S., Wu, J., Weiss, M.J., Bresnick, E.H.: GATA-1-dependent transcriptional repression of GATA-2 via disruption of positive autoregulation and domain-wide chromatin remodeling. Proc Natl Acad Sci U S A **100**(15), 8811--6 (2003). doi:10.1073/pnas.1432147100
9. Leonard, M., Brice, M., Engel, J., Papayannopoulou, T.: Dynamics of GATA transcription factor expression during erythroid differentiation. Blood **82**(4), 1071--1079 (1993). http://www.bloodjournal.org/content/82/4/1071.full.pdf
10. Bresnick, E.H., Lee, H.-Y., Fujiwara, T., Johnson, K.D., Keles, S.: GATA switches as developmental drivers. J Biol Chem **285**(41), 31087--93 (2010). doi:10.1074/jbc.R110.159079
11. Doré, L.C., Chlon, T.M., Brown, C.D., White, K.P., Crispino, J.D.: Chromatin occupancy analysis reveals genome-wide GATA factor switching during hematopoiesis. Blood **119**(16), 3724--3733 (2012). doi:10.1182/blood-2011-09-380634. http://www.bloodjournal.org/content/119/16/3724.full.pdf
12. Su, M.Y., Steiner, L.A., Bogardus, H., Mishra, T., Schulz, V.P., Hardison, R.C., Gallagher, P.G.: Identification of biologically relevant enhancers in human erythroid cells. J Biol Chem **288**(12), 8433--44 (2013). doi:10.1074/jbc.M112.413260
13. Li, L., Freudenberg, J., Cui, K., Dale, R., Song, S.-H., Dean, A., Zhao, K., Jothi, R., Love, P.E.: Ldb1-nucleated transcription complexes function as primary mediators of global erythroid gene activation. Blood **121**(22), 4575--4585 (2013). doi:10.1182/blood-2013-01-479451. http://www.bloodjournal.org/content/121/22/4575.full.pdf
14. Love, P.E., Warzecha, C., Li, L.: Ldb1 complexes: the new master regulators of erythroid gene transcription. Trends in Genetics **30**(1), 1--9. doi:10.1016/j.tig.2013.10.001
15. Kassouf, M.T., Hughes, J.R., Taylor, S., McGowan, S.J., Soneji, S., Green, A.L., Vyas, P., Porcher, C.: Genome-wide identification of tal1's functional targets: Insights into its mechanisms of action in primary erythroid cells. Genome Research **20**(8), 1064--1083 (2010). doi:10.1101/gr.104935.110. http://genome.cshlp.org/content/20/8/1064.full.pdf+html
16. Kang, Y., Kim, Y.W., Yun, J., Shin, J., Kim, A.: KLF1 stabilizes GATA-1 and TAL1 occupancy in the human -globin locus. Biochimica et Biophysica Acta (BBA) - Gene Regulatory Mechanisms **1849**(3), 282--289 (2015). doi:10.1016/j.bbagrm.2014.12.010
17. Han, G.C., Vinayachandran, V., Bataille, A.R., Park, B., Chan-Salis, K.Y., Keller, C.A., Long, M., Mahony, S., Hardison, R.C., Pugh, B.F.: Genome-wide organization of gata1 and tal1 determined at high resolution. Mol Cell Biol **36**(1), 157--72 (2016). doi:10.1128/MCB.00806-15
18. ENCODE Project Consortium: An integrated encyclopedia of DNA elements in the human genome. Nature **489**(7414), 57--74 (2012). doi:10.1038/nature11247
19. Forbes, S.A.: On the local distribution of certain Illinois fishes: an essay in statistical ecology, vol. 7. Illinois State Laboratory of Natural History, ??? (1907)
20. Whyatt, D.J., deBoer, E., Grosveld, F.: The two zinc finger-like domains of GATA-1 have different DNA binding specificities. EMBO J **12**(13), 4993--5005 (1993)
21. Xu, Z., Meng, X., Cai, Y., Koury, M.J., Brandt, S.J.: Recruitment of the swi/snf protein BRG1 by a multiprotein complex effects transcriptional repression in murine erythroid progenitors. Biochem J **399**(2), 297--304 (2006). doi:10.1042/BJ20060873
22. Kim, S.-I., Bultman, S.J., Kiefer, C.M., Dean, A., Bresnick, E.H.: BRG1 requirement for long-range interaction of a locus control region with a downstream promoter. Proc Natl Acad Sci U S A **106**(7), 2259--64 (2009). doi:10.1073/pnas.0806420106
23. Hu, G., Schones, D.E., Cui, K., Ybarra, R., Northrup, D., Tang, Q., Gattinoni, L., Restifo, N.P., Huang, S., Zhao, K.: Regulation of nucleosome landscape and transcription factor targeting at tissue-specific enhancers by BRG1. Genome Res **21**(10), 1650--8 (2011). doi:10.1101/gr.121145.111
24. Lan, X., Witt, H., Katsumura, K., Ye, Z., Wang, Q., Bresnick, E.H., Farnham, P.J., Jin, V.X.: Integration of Hi-C and ChIP-seq data reveals distinct types of chromatin linkages. Nucleic Acids Res **40**(16), 7690--704 (2012). doi:10.1093/nar/gks501
25. Ong, C.-T., Corces, V.G.: Ctcf: an architectural protein bridging genome topology and function. Nat Rev Genet **15**(4), 234--246 (2014)

26. Brookes, E., Pombo, A.: Modifications of RNA polymerase ii are pivotal in regulating gene expression states. EMBO Rep **10**(11), 1213--9 (2009). doi:10.1038/embor.2009.221
27. Simon, J.A., Kingston, R.E.: Occupying chromatin: Polycomb mechanisms for getting to genomic targets, stopping transcriptional traffic, and staying put. Molecular Cell **49**(5), 808--824. doi:10.1016/j.molcel.2013.02.013
28. Schultz, D.C., Ayyanathan, K., Negorev, D., Maul, G.G., Rauscher, F.J. 3rd: Setdb1: a novel kap-1-associated histone h3, lysine 9-specific methyltransferase that contributes to hp1-mediated silencing of euchromatic genes by krab zinc-finger proteins. Genes Dev **16**(8), 919--32 (2002). doi:10.1101/gad.973302
29. Frietze, S., O'Geen, H., Blahnik, K.R., Jin, V.X., Farnham, P.J.: Znf274 recruits the histone methyltransferase setdb1 to the 3' ends of znf genes. PLoS One **5**(12), 15082 (2010). doi:10.1371/journal.pone.0015082
30. Borck, G., Hög, F., Dentici, M.L., Tan, P.L., Sowada, N., Medeira, A., Gueneau, L., Thiele, H., Kousi, M., Lepri, F., Wenzeck, L., Blumenthal, I., Radicioni, A., Schwarzenberg, T.L., Mandriani, B., Fischetto, R., Morris-Rosendahl, D.J., Altmüller, J., Reymond, A., Nürnberg, P., Merla, G., Dallapiccola, B., Katsanis, N., Cramer, P., Kubisch, C.: Brf1 mutations alter RNA polymerase iii-dependent transcription and cause neurodevelopmental anomalies. Genome Res **25**(2), 155--66 (2015). doi:10.1101/gr.176925.114
31. Woon Kim, Y., Kim, S., Geun Kim, C., Kim, A.: The distinctive roles of erythroid specific activator GATA-1 and nf-e2 in transcription of the human fetal -globin genes. Nucleic Acids Res **39**(16), 6944--55 (2011). doi:10.1093/nar/gkr253
32. Vakoc, C.R., Letting, D.L., Gheldof, N., Sawado, T., Bender, M.A., Groudine, M., Weiss, M.J., Dekker, J., Blobel, G.A.: Proximity among distant regulatory elements at the beta-globin locus requires GATA-1 and FOG-1. Mol Cell **17**(3), 453--62 (2005). doi:10.1016/j.molcel.2004.12.028
33. Kim, S.-I., Bultman, S.J., Kiefer, C.M., Dean, A., Bresnick, E.H.: BRG1 requirement for long-range interaction of a locus control region with a downstream promoter. Proceedings of the National Academy of Sciences **106**(7), 2259--2264 (2009). doi:10.1073/pnas.0806420106. http://www.pnas.org/content/106/7/2259.full.pdf
34. Blattler, A., Yao, L., Wang, Y., Ye, Z., Jin, V.X., Farnham, P.J.: ZBTB33 binds unmethylated regions of the genome associated with actively expressed genes. Epigenetics & chromatin **6**(1), 13 (2013)
35. Yun, W.J., Kim, Y.W., Kang, Y., Lee, J., Dean, A., Kim, A.: The hematopoietic regulator tal1 is required for chromatin looping between the -globin lcr and human -globin genes to activate transcription. Nucleic Acids Research **42**(7), 4283--4293 (2014). doi:10.1093/nar/gku072. http://nar.oxfordjournals.org/content/42/7/4283.full.pdf+html
36. Frietze, S., O'Geen, H., Blahnik, K.R., Jin, V.X., Farnham, P.J.: Znf274 recruits the histone methyltransferase setdb1 to the 3 ends of znf genes. PLoS ONE **5**(12), 1--15 (2010). doi:10.1371/journal.pone.0015082
37. Jaccard, P.: Etude comparative de la distribution florale dans une portion des Alpes et du Jura, vol. 37. Impr. Corbaz, ??? (1901)
38. Blobel, G.A., Nakajima, T., Eckner, R., Montminy, M., Orkin, S.H.: Creb-binding protein cooperates with transcription factor GATA-1 and is required for erythroid differentiation. Proceedings of the National Academy of Sciences **95**(5), 2061--2066 (1998). http://www.pnas.org/content/95/5/2061.full.pdf
39. Letting, D.L., Rakowski, C., Weiss, M.J., Blobel, G.A.: Formation of a tissue-specific histone acetylation pattern by the hematopoietic transcription factor GATA-1. Molecular and Cellular Biology **23**(4), 1334--1340 (2003). doi:10.1128/MCB.23.4.1334-1340.2003. http://mcb.asm.org/content/23/4/1334.full.pdf+html
40. Wang, X., Nagl, N.G., Wilsker, D., Van Scoy, M., Pacchione, S., Yaciuk, P., Dallas, P.B., Moran, E.: Two related ARID family proteins are alteRNAtive subunits of human swi/snf complexes. Biochemical journal **383**(2), 319--325 (2004). doi:10.1042/BJ20040524. http://www.biochemj.org/content/383/2/319.full.pdf
41. Drasgow, F.: Polychoric and polyserial correlations. Encyclopedia of statistical sciences (1988)
42. Olsson, U.: Maximum likelihood estimation of the polychoric correlation coefficient. Psychometrika **44**(4), 443--460 (1979)
43. Pearson, K.: Mathematical contributions to the theory of evolution. vii. on the correlation of characters not quantitatively measurable. Philosophical Transactions of the Royal Society of London. Series A, Containing Papers of a Mathematical or Physical Character **195**, 1--405 (1900)
44. Grebien, F., Kerenyi, M.A., Kovacic, B., Kolbe, T., Becker, V., Dolznig, H., Pfeffer, K., Klingmuller, U., Muller, M., Beug, H., Mullner, E.W., Moriggl, R.: Stat5 activation enables erythropoiesis in the absence of EpoR and Jak2. Blood **111**(9), 4511--4522 (2008)
45. Wierenga, A.T.J., Vellenga, E., Schuringa, J.J.: Down-regulation of GATA1 uncouples STAT5-induced erythroid differentiation from stem/progenitor cell proliferation. Blood **115**(22), 4367--4376 (2010)
46. Cantor, B. A., Orkin, S.H.: Transcriptional regulation of erythropoiesis: an affair involving multiple partners. Oncogene **21**(21), 3368--3376 (2002)
47. Kerenyi, M.A., Orkin, S.H.: Networking erythropoiesis. J Exp Med **207**(12), 2537--41 (2010). doi:10.1084/jem.20102260
48. Xu, J., Shao, Z., Glass, K., Bauer, D.E., Pinello, L., Van Handel, B., Hou, S., Stamatoyannopoulos, J.A., Mikkola, H.K.A., Yuan, G.-C., Orkin, S.H.: Combinatorial assembly of developmental stage-specific enhancers controls gene expression programs during human erythropoiesis. Developmental Cell **23**(4), 796--811. doi:10.1016/j.devcel.2012.09.003
49. Cheng, Y., Ma, Z., Kim, B.-H., Wu, W., Cayting, P., Boyle, A.P., Sundaram, V., Xing, X., Dogan, N., Li, J., Euskirchen, G., Lin, S., Lin, Y., Visel, A., Kawli, T., Yang, X., Patacsil, D., Keller, C.A., Giardine, B., Mouse ENCODE Consortium, Kundaje, A., Wang, T., Pennacchio, L.A., Weng, Z., Hardison, R.C., Snyder, M.P.: Principles of regulatory information conservation between mouse and human. Nature **515**(7527), 371--5 (2014). doi:10.1038/nature13985
50. Whyte, W.A., Orlando, D.A., Hnisz, D., Abraham, B.J., Lin, C.Y., Kagey, M.H., Rahl, P.B., Lee, T.I., Young, R.A.: Master transcription factors and mediator establish super-enhancers at key cell identity genes. Cell **153**(2), 307--319. doi:10.1016/j.cell.2013.03.035
51. Hnisz, D., Abraham, B.J., Lee, T.I., Lau, A., Saint-André, V., Sigova, A.A., Hoke, H.A., Young, R.A.: Super-enhancers in the control of cell identity and disease. Cell **155**(4), 934--47 (2013).

doi:10.1016/j.cell.2013.09.053
52. Fox, J.: Polycor: Polychoric and Polyserial Correlations. (2016). R package version 0.7-9. https://CRAN.R-project.org/package=polycor
