# Supplementary material for: GSuite HyperBrowser: integrative analysis of dataset collections across the genome and epigenome
Source: Gigascience. 2017 Apr 27;6(7):1–12. doi: 10.1093/gigascience/gix032 (PMC5493745; doi:10.1093/gigascience/gix032)
Supplement: Additional File 3: — A text document with a detailed specification of the GSuite file format (PDF format, 65 KB). [file gix032_Additional_file_3.pdf]

Additional file 3: Specification of the GSuite file format

Simovski et al., "GSuite HyperBrowser: integrative analysis of dataset collections across the genome and epigenome"

GSuite version: 0.9

Contents
----

* Introduction and background
    - Overview
    - Suites of tracks
    - Location of tracks
    - Preprocessing of tracks into a binary format (BTrack)
    - Track types
* Example GSuite files
* Syntax of the GSuite format
    - Introduction to the line types
    i. Empty lines
    ii. Comment lines
    1. Header lines
    2. Column specification line
    3. Track lines
* References
* Change log

Introduction and background

Overview

GSuite is a simple tabular text format for use in specifying a suite (ie. a set or collection) of genomic annotation tracks (simply called tracks in this document). A GSuite file does not contain any genomic data as such, but provides metadata necessary to locate the track contents, info on whether the track has been preprocessed in a manner suitable for analysis (see the BTrack file format), some basic information on how to analyze the data (see the track type concept), as well as the reference genome build that the track coordinates are based upon. In addition to this, the user may add as many custom metadata columns he/she needs.

Suites of tracks

Central to the concept of track suites is the idea that tracks which take part in a GSuite file should be somewhat related in contents and format. Although the GSuite format allows heterogeneous tracks to be banded together in a single file, such files will typically not be useful for analysis purposes, as one would almost always need to restrict the contents and/or format as required by the analysis tools. For instance, a tool that finds the intersection of base pairs covered by all tracks in a suite would require all tracks to be of type "points" or "segments", not "function", as tracks of that type cover all base pairs (see section Track Types below for more info). For this reason, the GSuite file format specifies a set of four header variables that could (and should) be stated in the beginning of the file. These header variables function as a summary over the tracks in the file, providing a specific value if all the tracks are in accordance with each other. If the different tracks varies on this particular aspect, the header variable is set to the reserved keyword "multiple". This typically indicates that the collection of tracks is not yet focused enough to be usable as a suite of tracks for analysis purposes.

Location of tracks

In order to analyze multiple tracks, one obviously needs to acquire such tracks. Some tracks one might have acquired directly from sequencing endeavors (e.g. ChIP-seq peaks), but often one needs to fetch such tracks from public repositories and databases such as those provided by the ENCODE [1] and Roadmap Epigenomics [2] projects. GSuite supports the specification of suites of tracks before the actual track files has been retrieved from a server. In such cases, the location of the tracks are termed "remote" and the GSuite file would typically contain an HTTP or FTP address to the remote location. Tracks

that have been retrieved and is stored at the same place as the GSuite file are termed as "local".

Preprocessing of tracks into a binary format (BTrack)

As part of the implementation of an analysis tool, one would typically need a track to be translated, or preprocessed, into a binary format before analyses takes place, as this greatly improves analysis speed. Often this is done behind-the-scenes inside the analysis tool. In the GSuite format, however, the concept of preprocessed binary versions of a track has been included explicitly as part of the format. The reason for this is that preprocessing typically takes some time per track, and when one works with multiple tracks (often hundreds) this step will thus consume a significant amount of time. Carrying out the preprocessing step as a one-time process, instead of every time one runs an analysis tool, will thus save much time for the user. Analysis tools therefore typically require the tracks in a GSuite to be preprocessed in advance.

Preprocessing of a GSuite file results in the tracks being stored in the BTrack format. BTrack is a binary format for genomic tracks that allows for fast retrieval and efficient analyses by the storage of data columns as numeric arrays. An analogue to the BTrack format in the domain of sequence alignment is the BAM format, which is a binary version of the textual SAM format. BTrack is thus the binary version of the previously published GTrack format [3].

BTrack is the new name for the previously unnamed internal track storage format used in the Genomic HyperBrowser [3,4]. The BTrack format has seen several major updates as part of the HyperBrowser code base, and will now soon be released as a separate binary format that allows multiple tracks to be stored in a single binary file (currently unpublished). The GSuite format is intimately linked to the BTrack format, as a BTrack file would be able to store both a GSuite file together with the actual track contents.

Track types

The concept of track types has been examined in detail in a previous publication [2]. Briefly, a track type is a characterization of a the geometrical/mathematical properties of a track. A track is typically envisioned as data somehow located along the DNA sequence of a particular reference genome. The simplest track type is "points", which refer to single

base pairs scattered along the genome, e.g. SNPs. "Segments" are the more common ones, which represents regions of the DNA, e.g. genes. With the addition of values and/or cross-genomic links, a total of 15 track types was delineated in [3]. The main usage scenario of track types is to limit which tracks it makes sense to use as input to a particular analysis tool. For example, an analysis of the base pair overlap of two tracks would typically require the tracks to be of type "segments". When it comes to the analysis of multiple tracks, one would typically require the tracks to be analyzed to be of the same track type. The GSuite format thus supports "track type" as one of the main header variables (see below). The following is a list of all the supported 15 track types, as delineated in [3]:

Points (P)
Valued Points (VP)
Segments (S)
Valued Segments (VS)
Genome Partition (GP)
Step Function (SF)
Function (F)
Linked Points (LP)
Linked Valued Points (LVP)
Linked Segments (LS)
Linked Valued Segments (LVS)
Linked Genome Partition (LGP)
Linked Step Function (LSF)
Linked Function (LF)
Linked Base Pairs (LBP)

Example GSuite files

Before going into the details of the GSuite format, one should be able to get a quick overview of the format by looking at these example files:

```
# Example 1: List of URLs

http://www.server.com/path/to/file.bed
http://www.server.com/path/to/file2.bed
http://www.server2.com/path/to/other_file.bed
ftp://www.server3.com/path/to/new_file.wig
```

```
# Example 2: List of URLs with header lines
```

```
##location: remote
##file format: primary
##track type: segments
##genome: hg38
http://www.server.com/path/to/file.bed
http://www.server.com/path/to/file2.bed
http://www.server2.com/path/to/other_file.bed
ftp://www.server3.com/path/to/new_file.gff


# Example 3: List of URLs with header lines, comments, extra columns, and
# GSuite-specific URI (Uniform Resource Identifier) schemes

##location: multiple
##file format: multiple
##track type: segments
##genome: hg38
###uri   title   p-values
http://www.server.com/path/to/file.bed   track_1   0.002
http://www.server.com/path/to/file2.bed   track_2   0.1
http://www.server2.com/path/to/other_file.bed   track_3   1.0
ftp://www.server3.com/path/to/new_file.gff   track_4   0.8
galaxy:/abcd1234abcd;bed   track_5   0.012
hb:/my/track/name   track_6   .
```

Syntax of the GSuite format

Introduction to the line types

GSuite is a tabular text file format. All GSuite filenames should end with ".gsuite". The GSuite format consists of 5 different line types, distinguished by the leading characters and numbered here by order of appearance in the file:

i. Empty lines
ii. Comment lines
1. Header lines
2. Column specification line
3. Track lines

Note: The arabic number preceding each line type defines the order in which the lines must be present. I.e. column specification must follow the header

lines. Roman numbers indicate comments and emtpy lines, which may be present anywhere.

----
i. Empty lines

- Leading characters: none

- Syntax:

  only whitespace characters (space, tab, newline, return)

- Usage: optional

- Description:

  Empty lines are allowed anywhere in the GSuite file. These will be ignored by the parsers

----
ii. Comment lines

- Leading characters: # (a single hash character)

- Example:

  # this is a comment

- Usage: optional

- Description:

  Comments are allowed anywhere and will be ignored by parsers. Note that a comment line following a track line is considered to be a comment for that track and can for instance be used by tools that creates GSuite files to present track-specific error messages to the user.

----
1. Header lines

- Leading characters: ##

- Syntax:

    ##variable:[ ]*value

    where
      variable = Header variable name
      [ ]* = Optional space characters
      value = Header variable value

- Example:

    ##location: local
    ##file format: preprocessed
    ##track type: segments
    ##genome: hg38

- Usage:

  optional in an input GSuite file, but auto-generated when a GSuite is created as output from a tool

- Description:

  A header variable contains information that relates to the whole of the GSuite file, and is thus a summary over all the tracks in the file. The header variables names are limited to a set of reserved keywords, each with a restricted set of values. The header variables are related to reserved columns of the track lines (see the section "Column specification line" below).

- Parser notes:

  If a header variable is missing, it will be auto-generated from the track lines. If a header variable is present, but with a value that is inconsistent with the track lines, the parser will return an error. Note that all header variable lines except for the "genome" variable allow a mix of lower- and uppercase characters.

  The following logic for the values "unknown" and "multiple" will hold for all header variables:

  Unknown: if at least one track has "unknown" as it value, the value of the GSuite header variable will also be "unknown", regardless of the values for the other tracks.

  Multiple: if at least one track has a different value than the others, the

value of the GSuite header variable will be "multiple" (unless the value for one of the tracks is "unknown", in which case that keyword takes precedence).

Reserved header variable names

- Location:

  Specifies whether the data contents of all tracks in the GSuite are found at remote locations on the Internet, or if they have been downloaded locally to the service parsing the GSuite file (see section "Location of tracks" above). Note that the service parsing the GSuite may itself be located on e.g. a web server, but the tracks of the GSuite is still considered as local if they are on the same server as the service.

  The location header is a summary of the different types of URI schemes present in the "uri" column in the track lines (see the section "Column specification line" below). All supported types of URIs are thus defined as either remote or local.

  Allowed values: unknown, remote, local, multiple

- File format:

  Specifies whether all tracks have been preprocessed into the binary format BTrack, which is a prerequisite for most analysis tools. The "file format" header variable is a summary of the contents of the "file_format" column in the track lines (see the section "Column specification line" below).

  Allowed values: unknown, primary, preprocessed, multiple

- Track type:

  Specifies the track type common for all the tracks in the GSuite file, if any. See the section "Track types" above for more information. The "track type" header variable is a summary of the contents of the "track_type" column in the track lines (see the section "Column specification line" below).

  Note that if the track types of the tracks are different, but based upon the same basic type, the common track type of the GSuite file is set to the simplest track type that can used to describe all tracks, if any. E.g. if two tracks have the types "valued segments" and "linked segments",

respectively, the track type of the GSuite file is "segments". If there is no such simple track type, the keyword "multiple" is used.

Allowed values: unknown, points, valued points, segments, valued segments, genome partition, step function, function, linked points, linked valued points, linked segments, linked valued segments, linked genome partition, linked step function, linked function, linked base pairs, multiple

- Genome:

Specifies the reference genome for all the tracks in the GSuite file. The "genome" header variable is a summary of the contents of the "genome" column in the track lines (see the section "Column specification line" below. The actual keyword for the genome build is dependent on the implementation of the analysis tools that will make use of the information. The GSuite format accepts any string as the genome.

Allowed: unknown, multiple, any other string specifying a reference genome

----
2. Column specification line

- Leading characters: ###

- Syntax:

```
###col1  col2  col3...
```

where
  col1, col2, col3 = Column names
  " " = tab character

- Example:

```
###uri  title  file_format  track_type  genome  description  p-value
```
  (with tabs instead of spaces)

- Default value:

```
###uri
```

- Usage:

Optional, but if not defined the column specification line retains the

default value. This means that a list of URI's is a valid GSuite file.

- Description:

  The column specification line is a tab-separated list of column names. The GSuite specification defines a set of five reserved column names:

    uri, title, file_format, track_type, genome

  In addition, any number of custom column names can be specified.

- Parser notes:

  Column names are treated as case insensitive. All column names must also be unique. The columns can be ordered in any way, but it is recommended for readability to use "uri" and "title" as the first two rows, if defined.

Reserved column variable names

- URI:

  A unique identifier following the Universal Resource Identifier format [5]. GSuite supports the following standard URI schemes for data residing at a remote location:

    ftp, http, https, rsync

  Examples:

    ftp://ftp.server.com/path/to/file.bed
    http://www.server.com:8080/index?filename=track.wig
    rsync://server.com/path/to/file

  For local files, the standard "file" URI scheme is also supported, e.g.:

    file:///path/to/file/bed

  Note that the "file" scheme does not support files residing other places than "localhost". The host part of the URI is thus uneeded, hence the triple '/' characters.

  Two more specifically specified URIs schemes are supported by GSuite:

    "galaxy" and "hb"

The "Galaxy" scheme uniquely identifies a Galaxy dataset, but currently only works for the local installation of the Galaxy analysis framework that is set up with GSuite support, i.e. one cannot (yet) provide an URI to a remote Galaxy installation [6]. The syntax is as follows:

galaxy:/dataset_key[/directory/structure/to/file]

Multiple files can be stored within one Galaxy history element using the directory structure syntax.

The "HB" scheme identifies a track stored as the BTrack format within the local installation of GSuite HyperBrowser. The syntax is as follows:

hb:/track/name/hierarchy

Note that for all the URI schemes except the "HB" one, GSuite supports the additional specification of file suffix after a semicolon, as in this example:

ftp://ftp.server.com/path/to/file;bed

This usable if the file path itself does not contain the suffix, and hence does not contain any information on the actual file format of the track.

- Parser notes:

  Note that services available from e.g. the web should disable the "file" scheme, as this is inherently insecure.

- Title:

The title of the track, as specified by the user. Each track title must be unique within a specific GSuite, so that one may use the title as a key to uniquely reference specific tracks in a GSuite.

Allowed values: *any*

- File_format:

Specifies whether the track has been preprocessed into the binary format BTrack or not, as described in the section "Header lines" above.

If the GSuite parser understands the file suffix to be an un-preprocessed format, file format is automatically set to "primary". Similarly, tracks in the BTrack format (including those with "HB" as URI) automatically gets "preprocessed" as "file_format".

Allowed values: unknown, primary, preprocessed

Default value: unknown

- Track_type:

  Specifies the track type of the track, as described in the section "Header lines" above. If the track is preprocessed into a BTrack file, the value of the "track_type" is automatically collected from the BTrack file(s) themselves.

  Allowed values: unknown, points, valued points, segments, valued segments, genome partition, step function, function, linked points, linked valued points, linked segments, linked valued segments, linked genome partition, linked step function, linked function, linked base pairs

  Default value: unknown

- Genome:

  Specifies the reference genome build used as basis of the track, as described in the section "Header lines" above.

  Allowed: unknown, any other string specifying a reference genome

  Default value: unknown

- Custom columns

  Any number of custom columns can be added. Any string can be used as value for each track, so there are little or no rules on the content defined within the GSuite format. Missing values for custom columns are denoted with the period character: '.'

Optional columns

If the value in the "file_format" column is the same for all tracks in a GSuite, the column can be removed, leaving only the value of the "file format" header variable to speak for all individual tracks. The same logic holds also for the columns "track_type" and "genome".

----
3. Track lines

- Leading characters: none

- Syntax:

```
val1  val2  val3...
```

where
val1, val2, val3 = column values
" " = tab character

- Example:

```
###uri                                    title           p-value
http://www.server.com/path/to/file.bed   My cool track   0.00013
```

(with tabs instead of spaces)

- Usage

Track lines are optional. If no track lines are specified, the GSuite file represents an empty collection of tracks.

- Description

Each track is specified as a tab-separated list of metadata values, as defined by the column specification line. See the section "Column specification line" for a more detailed discussion on the allowed values.

References
----

[1] ENCODE Project Consortium. "An integrated encyclopedia of DNA elements in the human genome." Nature 489.7414 (2012): 57-74.
[2] Kundaje, Anshul, et al. "Integrative analysis of 111 reference human epigenomes." Nature 518.7539 (2015): 317-330.
[3] Gundersen, Sveinung, et al. "Identifying elemental genomic track types and representing them uniformly." BMC Bioinformatics 12.1 (2011): 1.
[4] Sandve, Geir K., et al. "The Genomic HyperBrowser: inferential genomics at the sequence level." Genome biology 11.12 (2010): 1-12.
[5] Uniform Resource Identifier (URI): Generic Syntax (https://tools.ietf.org/html/rfc3986)

[6] Goecks, Jeremy, Anton Nekrutenko, and James Taylor. "Galaxy: a comprehensive approach for supporting accessible, reproducible, and transparent computational research in the life sciences." Genome Biol 11.8 (2010): R86.

Change log
----

v0.1 - 2015.07.06:

* Initial version of the GSuite specification document.

v0.2 - 2016.07.06:

* Fixed typos and cleaned up text several places. Ready for initial submission og the GSuite HyperBrowser manuscript.
