# Supplementary material for: GSuite HyperBrowser: integrative analysis of dataset collections across the genome and epigenome
Source: Gigascience. 2017 Apr 27;6(7):1–12. doi: 10.1093/gigascience/gix032 (PMC5493745; doi:10.1093/gigascience/gix032)
Supplement: Response-to-reviewer-comments_Original-Submission.pdf [file gix032_Response-to-reviewer-comments_Original-Submission.pdf]

To the editor:
Please find attached the revised manuscript and point-by-point responses to the reviewers' comments. Regarding conformance with journal style, we were unfortunately not successful in using the new provided templates. An issue related to a certain font (msyr) prevented us in compiling the manuscript on several different Latex environments. We have therefore kept the latex formatting style that we used for our initial submission.

General comment to reviewers:
We greatly appreciate the time and focus the reviewers dedicated to our manuscript. We found the comments highly constructive and acted upon them to the best of our abilities in the time constraint given. We sincerely believe that the reviewers' inputs have helped us to significantly improve and finalize the manuscript. Clearly, this work will be continuously ongoing, and further development is expected with the increased amount of user interaction that hopefully will come with the publication of this paper.

Response to reviewer 1

General comment: I'm totally agree with a lot of statement of the article as considering the fact that "a new layer of computational methodology is needed" and I like the idea of "next generation tools" as the fact to consider first the biological question. Unfortunately, I found that the manuscript is quite difficult to read, for sure because some concept categorizations are quite hard to follow for someone like me (considering classes of multiplicity, algorithms or statistics), but maybe some improvement in the presentation of the concepts, better figures, schemas, can help the reader better catch and understand the presented complexity and associated challenges.

Response:
As detailed in our response to point 12 below, we have added several schematic illustrations in order to convey some of the core concepts in a more intuitive manner, including a schematic illustration of the classes of multiplicity. We have also tried to make various changes to the manuscript text, which we hope will contribute to improved readability.

1) You mention p6 "in this example, using the Forbes measure to assess co-occurrence resulted in a biologically very reasonable ranking of potentially TFs, whereas the Jaccard measure produced a ranking that appeared severely biased by the number of peaks in each track from the suite". I'm not an expert of Forbes and Jaccard measure, but I'm interested to know more. Can you go further and explain more deeply this finding and give the reasons of this statement?

Response:
We appreciate the interest in this issue, which we have also ourselves found very intriguing. During the revision, we have looked further into the effect of similarity measures, introducing also a third similarity measure (the tetrachoric correlation, see below) in our detailed analysis, and extensively updated Additional File 2 to provide a clearer account of the implications. As part of these updates, we have also tried to expand and clarify the discussion of these issues in the manuscript, specifically in the subsection "Illustrative example" and in the paragraph on "Contrasting multiplicity" in the subsection "Classes of multiplicity for analyses of track suites".
Briefly, when measuring the similarity between two tracks, it is natural to consider two independently generated tracks as a neutral baseline (being neither similar nor dissimilar). Both the Forbes coefficient and the tetrachoric correlation has such a well-defined neutral value (1 for Forbes, 0 for correlation), regardless of how many elements the tracks contain. However, even for independently generated tracks, the Jaccard index varies greatly depending on the number of elements in the tracks (as shown using simulated data in Section 2 of Additional File 2). Also, when ranking tracks representing real genomic data (experimental datasets for K562), we see a strong tendency for large tracks to be ranked at the top when using the Jaccard Index (Section 1 of Additional File 2). Therefore, we now explicitly conclude in the manuscript that we generally don't recommend use of the Jaccard Index in situations where tracks are to be contrasted/ranked.

We believe much more may be explored regarding the properties of such similarity measures, but feel we have now taken it as far as we can within the scope of the present manuscript. Our main aim is to make readers aware that the mathematical details of the analysis may have a large influence on the final results.

2) You mention p10 that GSuite HyperBrowser is prepared for future extensions in a variety of dimensions. Considering following test relative comments, and notably the problem associated to the use of an old Galaxy instance, I'm quite afraid this flexibility will be hard to maintain. Can you argue on that point? I'm totally agree with the statement about the fact that current statistics only relate to pure location data and I encourage authors to go ahead on the huge challenge behind this statement!

Response:
As detailed in our response to point 6 below, we have in the revision round taken considerable efforts to streamline our development process and prepare our system for future Galaxy updates. Hopefully this shows our determination and ability to support our claims of extensibility and robustness of the system.
The current system architecture allows statistical measures based on features beyond pure location data (values for intensities, categories of elements, connections between elements etc.) to be plugged into the existing tools easily. Considering the broadness of the GSuite HyperBrowser system, we found it useful to restrict the scope of the present manuscript to pure location data, but we are strongly interested in further exploring analyses with extended scope.

3) Concerning "consistent terminology for track metadata", can the authors give some insight, maybe regarding the use of ontology or web semantic approaches?

Response:
We appreciate the interest into this issue. As argued in the paper, we view that the creation of a "consistent terminology for track metadata" would be very important in order to ease track data integration. Such work should, in our opinion, be organized as a community effort, and we have added a sentence to the manuscript about this: "Ideally, this should be organized as a community effort to ensure international uptake."
We are currently approaching the Elixir ESFRI infrastructure to take the lead on the creation or adaption of an ontology for track metadata, ideally in collaboration with the major entities providing track data. A possibility would be to make use of, and possibly extend, existing ontologies, such as the EDAM ontology. The standardization of metadata based upon ontologies is a natural first step. One could further easily envision the advantages that the application of semantic web technologies would bring to the information retrieval process. Semantic web is also an area of competence within the Elixir organization. As this idea is currently in its infancy, we feel that it is beyond the scope of the present manuscript to go into more details beyond the above mentioned addition.

4) Figures appear to be in a quite bad quality, but maybe this is just because of the "review formatting"?

Response:
We have prepared the figures in high resolution, and assume the bad quality is due to the review formatting. Given the manuscript proceeds towards publication, we would contact the copyeditors to make sure that the figures are represented appropriately.

5) Reference 21 shows some "???"
Response:
The issue has now been corrected.

6) A first general comment is related to the fact to customize a Galaxy instance. If I really like the idea of having a personal dedicated instance with advanced functionalities and better graphics, to my knowledge, all

deeply customized instance I have used, suffered many problems including update capabilities. I see that the Genomic Hyperbrowser is not based on a recent Galaxy instance (and maybe a quite very old one). This is maybe not totally due to this customization layer, but often, customization leads to a bigger cost related to instance update. This is really not a good thing when you work with a tool like Galaxy who is often updated and this can lead to security problems and difficulties to use wonderful new functionalities as Interactive tours mentioned above or the scratchbook view.

Response:
We completely agree with this point. We set up our customized Galaxy variant in mid 2008, and have since experienced update challenges along the lines the reviewer is suggesting. As a lot of our planned and ongoing in-house development relies on the HyperBrowser platform, we are ourselves in need for timely updates. To be better able to keep up with Galaxy updates, we have thus during the revision period fundamentally changed and streamlined our development process. As part of this, we have also moved to Galaxy version 2016.01, and plan to update to 2016.10 very soon.
Specifically, we have moved all the HyperBrowser code into GitHub, and have switched from a patch-based Galaxy customization approach to an approach built around git mechanics. We have also separated our customized functionality into two layers. Most customizations are now part of a separate Galaxy fork named Galaxy ProTo, which contains the code for our Python-based tool development solution (first layer). ProTo has no specific functionality related to GSuite or statistical genome analysis, and is something we will try to push in its own right. We are confident that this will be easy to maintain, as the intrusions into Galaxy core code are few and small. A further (small) set of GSuite HyperBrowser-specific customizations is part of a GSuite HB repository, which is again forked from the ProTo repository (second layer). Our update process will thus be to at the first level pull Galaxy updates into the ProTo repository (fork of Galaxy), and after resolving any issues, pull ProTo updates further onto the GSuite HB repository (fork of ProTo). We are confident that this improved development process will make us able to keep up with Galaxy updates based on a limited effort. There will of course be some delays in the update process, and we might have a slightly slower update schedule than that of Galaxy, but we are confident that we will from now on be able to provide timely updates of the HyperBrowser source code.
Also note that we specifically handle discovered Galaxy security problems through receiving email notifications from the Galaxy team ahead of full public disclosure, and apply the accompanying patches promptly, without having to update the rest of the code base at the same time.

7) From the main page, I met difficulties to access screencasts from the "Brief demos" section. Apparently, the https://screencast.uninett.no/relay/ansatt/geirksauio.no/... Links were broken at the moment I want to access it. Switching to the "Basic Mode" section, I really appreciated the use of the biological question as a starting point! I think this is something we have to use more generally when presenting bioinformatics tools functionalities.

Response:
We are sorry about this. Initially we put the videos on a nationally provided server for screencasts. Unfortunately it turned out to not be perfectly reliable. We have now moved the screencasts to Vimeo (which is what the Galaxy project is also using), thus making access more reliable. We appreciate the positive notice regarding our choice to start with the biological questions.

8) Maybe the use of Galaxy "interactive tour" functionality (https://usegalaxy.org/tours) will be a more accurate manner to present GSuite concepts?

Response:
It is an interesting idea that we didn't consider. We agree that it will be a nice addition, especially appreciated by experienced Galaxy users. That said, we provide help text, screencasts, and the interactive basic mode of operation. The added figures should clarify GSuite concepts even further. We feel it might become too messy if we add Galaxy tours now, but we will definitely include them in future designs.

9) Executing the tool "Which tracks (in a suite) coincide most strongly with a separate single track?", outputs are "customhtml" or "gsuite" data types. Again, as the Galaxy instance is not up-to-date, I can understand the use of html content powered by Highcharts. But using html and moreover customhtml format to present a table and associated graph don't appear the best manner to do for me. I always prefer to have the raw table, and here, at least, a possibility will be to propose the raw table in classical tabular format + an advanced html output. An alternative to your "customhtlm" output can be a report as for MultiQC (http://cloud-26.genouest.org/datasets/aa57f14b92934351/display/?preview=True). Concerning graphs, with an up-to-date Galaxy instance, it will be possible to generate it from the "Graph Visualization" functionality. Another way to have a dedicated visualization functionality can be the creation of a "real" Galaxy visualization (as for Trackster, Philoviz,…).

Response:
We agree that it is usually preferable to use existing Galaxy functionalities. The reason we decided to use Highcharts is the interactivity and flexibility it offers. Our intention is that the output of an analysis constitutes a standalone report. The customized result tables are part of it. We recognize that it is an oversight not to provide the raw data behind each result table, and we have now added this functionality. This will enable the user to use the standard Galaxy visualizations. Additionally, inspired by this comment, we have put into our future plans to provide a visualization plugin for Galaxy that would support the presentation of the results that is offered now in the GSuite HyperBrowser.

10) It would be good to have the scratchbook functionality to avoid escaping the "Basic Mode tuto page" each time you want to see a dataset. After using Basic mode section related tuto, I have tested several tools as "Create a remote GSuite from a public repository" or "Convert GSuite tracks from remote to primary (Download the tracks to the server)".

Response:
This issue has been resolved by the Galaxy update done as part of the response to issue 6. The very useful scratchbook functionality is now available.

11) Trying to view a hidden "GSuite track storage" dataset lead me to this strange message: "This history element contains GSuite track data, and is hidden by default. If you want to access the contents of this GSuite, please use the tool: DOES_NOT_EXIST_YET " Is such a tool in development ?

Response:
This has now been corrected. The appropriate tool to use is "Export primary tracks from a GSuite to your history" and this is now correctly linked to in the text.

12) You write : "This example task can be seen as a concrete instance of the more general question "How does a query track overlap the different tracks of a suite?", where: The query track is a dataset of genomic features (e.g. the genomic locations of specific SNP variants); The suite of tracks (a GSuite instance) is a collection of related genomic feature datasets (e.g. a collection of datasets which map the areas of active chromatin, with each dataset describing the genomic regions of DNaseI accessibility for a separate cell type)." Maybe pictures are a better way to illustrate the query tracks / suite of tracks concept ?

Response:
We agree and have added several new figures to help readers/users grasp the concepts. The five tools under the menu "Statistical analysis of GSuites", corresponding to the five statistical questions defined in Additional File 1, now contain illustrations that represent the concepts. In the manuscript we have added the figure corresponding to the statistical question "Which tracks (in a suite) coincide most strongly with a

separate single track?". Additionally, in the manuscript we included a figure explaining the concepts behind multiplicity, and a screenshot figure of the interactive Basic Mode.

13) A little typo on Basic Mode / Analysis step / 1-c "supported file formats" : the hyperlink is not activated for entire sentence….

Response:
This issue has now been corrected.

Responses to reviewer 2

It is nice to see the abstractions over local vs remote data sources. However it seems that a number of tools (basically all tools?) only function on local data sources breaking that transparency. Exporting tracks is an example of this, it seems to just select a single line from a GSuite file, but due to the addition of features to that tool (previewing, format conversion) this cannot be abstracted to remote data sources.

The sentence "can represent data at a local or remote server" got me a bit excited for a format which behaves transparently despite data locality, but I recognise the rationale behind the implementation. Re-reading this paragraph, it seems this was just me jumping to conclusions. Some of these tools could perhaps be extended to work on both local and remote datasets with greater flexibility, but I would not say this is important for the publication of this paper.

Response:
We think it is a great idea and will try to follow up with implementation in the future. Our current approach is to develop a well functioning system with manual steps first. Now that it is in place, we would like to build an automated layer on top of it, that would figure out the needed intermediate steps to allow a GSuite of any format to be used as input. We believe we would then be able to essentially provide such transparency as suggested. As e.g. the selection of a remote GSuite in a statistical analysis will require the data to be downloaded before analysis, the running time will differ based on the selected type, but it could still be transparent with respect to functionality and way of selecting. Also, even if the intermediate steps are inferred automatically, we would explicitly report them to the user, to ensure that the user has a full overview of all operations that have been performed.

I remember the presentation at GCC, proto galaxy for tool development is quite interesting as a concept. For actually deploying these tools to local galaxies, however, it comes across as a negative that we need a specific, old version of galaxy to run these tools. The fact that these tools are not available as standard tools with galaxy XML tool definitions will significantly hinder adoption by the wider community.

Response:
We recognize the issues created by using an old Galaxy, and have therefore updated to a newer version. We have also invested significant effort to make further updates easily applicable by moving all the HyperBrowser code into GitHub, and switching from a patch-based Galaxy customization approach to an approach built around git mechanics. We have also separated our customized functionality into two layers. As a direct git fork of Galaxy, ProTo is the first layer of this customization, and has no specific functionality related to GSuite or statistical genome analysis. We are confident that it will be easy to maintain, as the intrusions into Galaxy core code are few and small. A further (small) set of GSuite HyperBrowser-specific customizations is part of a GSuite HB repository, which is again forked from the ProTo repository (second layer). Our update process will thus be to at the first level pull Galaxy updates into the ProTo repository (fork of Galaxy), and after resolving any issues, pull ProTo updates further onto the GSuite HB repository (fork of ProTo). We are confident that this improved development process will make us able to keep up with

Galaxy updates based on a limited effort. ProTo has also been designed so as to make a "silent" update to existing Galaxies possible.
We agree that the fact that these tools are not available as standard tools with galaxy XML tool definitions represents an obstacle for their adoption. At the same time, it is this same avoidance of static definitions that allows the very dynamic tool interfaces and the efficient development process of ProTo. Our idea is to promote ProTo as a complement to the standard Galaxy tools, for situations where either very dynamic tool interfaces are needed, or in prototyping contexts where rapid development of tool interfaces is critical. With our recently improved deployment process, we believe this may see wider adoption.

The authors have done a good job of making their work accessing on public repositories. It is really wonderful to see this!
Unfortunately it seems that the SVN repos listed in both the INSTALL.txt file provided and the downloads page (https://hyperbrowser.uio.no/hb/static/download.html) reject my access. (svn: E000111: Can't connect to host 'invitro.titan.uio.no': Connection refused) I would recommend fixing this before publication. Additionally the INSTALL.txt does not mention the need to create a config/LocalOSConfig.py file, this should be adjusted for clarity.
Response:
We are grateful to the reviewer for testing the availability of the source code. Unfortunately there have been some changes in the network infrastructure at our university, rendering the URL obsolete.
However, we have since the submission of the first draft moved our development to GitHub. The source code is directly available from the web at "https://github.com/hyperbrowser/genomic-hyperbrowser", either as an archived download, or via git cloning. The installation process has also been greatly simplified and we have updated the INSTALL.txt file accordingly.
The LocalOSConfig.py file is also now obsolete and has been replaced by additional settings in the 'config/galaxy.ini' file, which is the standard Galaxy configuration file.

The basic vs advanced mode is quite useful, especially for someone unfamiliar with the tools installed in your system. Having a short list of pre-configured analyses is a great thing.

Response:
We appreciate the positive note on the basic mode, it is our intention to ease the introduction to the system to new users.

Many of the features implemented by GSuite files also exist in more recent releases of Galaxy as dataset collections. The authors should consider replacing future versions of GSuite files with a set of custom datatypes supporting the same metadata, and taking advantage of the new collections to make comparisons within and across lists of files.

Response:
This is a very interesting point, and we have added a brief discussion about it to the manuscript: "The Galaxy system also includes a native way of representing multiple datasets, termed dataset lists/collections, which we consider mostly complementary to GSuite. A strong aspect of dataset lists is their tight integration with Galaxy tool execution, which allows any standard Galaxy tool to be executed iteratively on each dataset of a collection. Through its representation as a tabular text file, GSuite is interoperable across systems and can be easily manipulated using any tool or software that operates on tabular datasets, inside or outside the Galaxy system. Furthermore, GSuite supports the specification of custom metadata for each dataset in a collection, which is exploited extensively in our tools and example analyses. We believe a general integration of the GSuite format within the Galaxy system, including functionality for converting between GSuite and dataset lists, could improve the usability of both the GSuite HyperBrowser and the standard Galaxy platform."

We consider GSuite and Galaxy dataset collections as partly complementary, in that they have partly complementary purposes and advantages. After the GSuite system has seen its final form and gone public, we aim to contact the Galaxy team to discuss how to best integrate these two developments, both in terms of conversion tools and on exploiting their complementarity.
The core difference between GSuite and dataset collections is that a GSuite is simply a tabular text file of a particular format (i.e. a dataset in itself), while a dataset collection corresponds to a particular representation of a set of Galaxy history elements internally in a Galaxy database. This again points to the key advantages of each solution: - GSuite can represent datasets both within and outside of Galaxy (can represent history elements or files hosted at any server), it can be edited using a variety of text manipulation tools inside or outside Galaxy (can e.g. be exported to local disk, and then manipulated in spreadsheet software or by custom tools).
- Galaxy datasets are more tightly integrated with the Galaxy system and can be used in any tool expecting a single dataset (can automatically call a tool with each dataset of the collection and merge each resulting dataset to a new collection). The way of defining dataset collections from a history is also very intuitive and flexible.
Another main distinction is that the GSuite format allows the representation of any set of metadata attributes for each track in a collection, where such metadata can be easily used or produced by tools due to it being a simple tabular text format (Galaxy dataset collections does not presently allow metadata to be annotated per dataset in the context of a particular collection, and may only be defined based on file format, not based on the original source or other characteristics of a dataset).
In summary, Galaxy dataset collections appear more focused on iteratively applying a Galaxy tool on each dataset in a collection, while GSuite is more focused on operations considering the collection as a whole and operations modifying what is contained in a given collection. Furthermore, Galaxy dataset collections are more intimately integrated with the Galaxy system, while GSuite allows better integration with resources outside Galaxy and better communication between systems.

The second supplementary file lists two URLs that are not rendered properly: the three dashes have been converted to an emdash preventing easy access.

Response:
The URLs have now been corrected.

Responses to reviewer 3

I noted GSuite uses column based text file for metadata, of which I am not against, but It could be restrictive in case of adding extra information, this issue could be easily handled using JSON format.

Response:
We see the potential advantages of JSON in terms of hierarchical representation and flexibility with respect to attributes. It could also be particularly useful in contexts of direct javascript interaction or RESTful web service interfaces. As the tabular format is so extensively used in Galaxy, with good support for viewing and manipulating through various tools, we find it most suitable as a main format for the GSuite HyperBrowser. It provides easy overview and can even be edited inside a spreadsheet editor of choice. Also note that in contrast to formats based on hard-coded columns (like bed), GSuite allows the specification of any metadata column of interest through a simple column specification header line.

Also, it would be better if authors mentioned about visual bits of the software in manuscript, and some visual representation of statistical analytics would be a great addition.

Response:
We now explicitly mention the availability of visual result output and specific visual analysis tools in the manuscript, by adding the following formulations to the manuscript: "The web interface includes [..] results in the form of sortable tables and customizable plots [..]" and "A set of tools for assembly and customization of track collections (GSuites) lead up to a diverse range of tools for statistical and visual analysis of relations between a multiplicity of tracks."
We have also added several new schematic illustrations to both the web system and the manuscript, in order to visually communicate some main concepts. Specifically, we have added schematic illustrations of the statistical computations for five tools, as well as including one of these illustrations in the manuscript, and the remaining four in supplementary material. We have furthermore added to the manuscript a schematic illustration of our proposed classes of multiplicity, as well as a screenshot showing the interface of the basic mode. Taken together, we believe these additions have substantially improved the accessibility of our proposed concepts, and are thankful for this being suggested in the reviewer comments.

I noticed the file format for the output was unknown in some steps (GSuite - remote files, and while creating GSuite), when following example steps, it could be misleading for some users, but not a deal breaker.

Response:
We appreciate being noted about this issue, which had slipped through our quality control. The reason that many remote GSuites (and modifications of these) had "unknown" file format was that the system did not correctly recognize compressed files, e.g. files of type "filename.bed.gz". This has now been corrected.

Though GSuite HyperBrowser is available on GitHub, but link is not clearly mentioned in manuscript.

Response:
At the moment of submission the GitHub repository was still private, and the source code was published at http://dx.doi.org/10.5281/zenodo.58138. The GitHub repository has now been made available and can be accessed at https://github.com/hyperbrowser/genomic-hyperbrowser
