# Supplementary material for: GSuite HyperBrowser: integrative analysis of dataset collections across the genome and epigenome
Source: Gigascience. 2017 Apr 27;6(7):1–12. doi: 10.1093/gigascience/gix032 (PMC5493745; doi:10.1093/gigascience/gix032)
Supplement: Response-to-reviewer-comments_revision-1.pdf [file gix032_Response-to-reviewer-comments_revision-1.pdf]

Reviewers comments

In the "Classes of multiplicity for analyses of track suites" section, authors mentions figure3a, 3b ... instead of 4a, 4b, …
Moreover, on the Figure 4, there is no a), b), c) or d).

Answer: Thank you for noticing this - we failed to update the numbering when adding new figures in the major revision. It has now been fixed.

*Testing https://hyperbrowser.uio.no/hb/#!mode=basic (warning, as I have had very low internet connection, issues mentionned here are maybe due to this technical limitation and not to a Galaxy instance problem):
*In the basic mode section / - click here to load a sample track with Multiple Sclerosis-associated regions, expanded 10kb in both directions (you will be automatically redirected back to this page if you choose this option); Not automatically redirected to the mentionned page, but on the home page
*Same with 2b step - click here to load a sample GSuite of DNaseI accessibility for different cell types (you will be automatically redirected back to this page if you choose this option);

Answer: We have tested this ourselves (on Chrome, Firefox and Safari) and could not recreate the issue. Our best assumption is that it is due to the system being accessed at a previous time point where it was not working, and that this state has been preserved in the cache of the browser. Clearing the client's web browser cache would resolve it, and it should only be an issue for any users that accessed the system at that previous time point where it was not working (i.e. not an issue for new users). We have now also added proper expiry date headers (access plus 6 hours) for the browser cache, so such an issue should not reappear (neither this nor a similar one) .

*As you are using "customhtml" output to propose to the user to export raw data table, it seems that user can't easily create a workflow using this output table. Maybe it can be of interest to propose directly on the tool formular an option for a "classical" text export.

Answer: We have given this suggestion a long consideration. While we recognize the usefulness of automated Galaxy workflows, our tools are not currently designed to support them. The GSuite HyperBrowser tools are tightly connected to the Galaxy ProTo extension (https://github.com/elixir-no-nels/proto), also developed by our team. Galaxy ProTo offers an alternative way of creating web tools without the use of XML definitions; instead the tools are directly and dynamically defined by Python code. We find that the added flexibility for building highly dynamic interfaces where each subsequent input can be dependent on previous selections, creates richer and more effective analysis tools. For example, the tools currently include several dynamic options that reflect GSuite contents, something that allows the analysis to be interactively customized to the particular research scenario.

With GSuite HyperBrowser, we have been focused on analytical scenarios where the analysis will be influenced by the data collections and the accompanying metadata; hence, the exact workflow steps and parameter selections will usually to some degree be unique. At the same time, by allowing to modify or analyze a whole collection through execution of a single tool, we believe we greatly decrease the need for repeated computations.
