# Supplementary material for: GSuite HyperBrowser: integrative analysis of dataset collections across the genome and epigenome
Source: Gigascience. 2017 Apr 27;6(7):1–12. doi: 10.1093/gigascience/gix032 (PMC5493745; doi:10.1093/gigascience/gix032)
Supplement: Response-to-reviewer-comments_revision-2.pdf [file gix032_Response-to-reviewer-comments_revision-2.pdf]

Editorial Comment:
I think the data DOI Chris is showing you will be reference 32, so you'll need to then renumber the citations following it. Firstly in the declarations section you can remove the ethics and consent parts as they aren't needed for this type of paper. The supporting materials then can be split into two parts/subsections, with software first as “Availability and requirements” and then the code/data snapshot as “Availability of supporting data”. In this new subsection you just need to add the GigaDB DOI reference number [32] here. I would suggest you write something like:

Availability of supporting data
A snapshot of the version of the Gsuite HyperBrowser source code used in this paper is archived in the GigaScience database GigaDB [32]"

... and include the following citation of the dataset in your references:

32. Simovski, B; Vodak, D; Gundersen, S; Domanska, D; Azab, A; Holden, L; Holden, M; Grytten, I; Rand, K; Drablos, F; Johansen, M; Mora, A; Lund-Andersen, C; Fromm, B; Eskeland, R; Gabrielsen, O, S; Ferkingstad, E; Nakken, S; Bengtsen, M; Nederbragt, A, J; Thorarensen, H, S; Akse, J, A; Glad, I; Hovig, E; Sandve, G, K (2017): Gsuite HyperBrowser version 2.0b GigaScience Database. http://dx.doi.org/10.5524/100292

Answer:
The changes to the declaration sections have been made accordingly, and the dataset is cited and included into the references as suggested.
