# Supplementary material for: GSuite HyperBrowser: integrative analysis of dataset collections across the genome and epigenome
Source: Gigascience. 2017 Apr 27;6(7):1–12. doi: 10.1093/gigascience/gix032 (PMC5493745; doi:10.1093/gigascience/gix032)
Supplement: Reviewer-1-Report-(Original-Submission).pdf [file gix032_Reviewer-1-Report-(Original-Submission).pdf]

Reviewer Report

Title: "GSuite HyperBrowser: integrative analysis of dataset collections across the genome and epigenome"

Version: Original Submission **Date:** 10/14/2016

Reviewer name: Yvan Le Bras

Reviewer Comments to Author:

The article presents GSuite HyperBrowser, a web platform dedicated to the integrative analysis of dataset collections across the genome and epigenome. Authors introduce the lack of universal methodology to analyse genome-wide, cell-type-specific profiles for various features. They present GSuite HyperBrowser, the first open-source solution proposing such analytical methodology across the genome and epigenome using Galaxy. The article is well written and I would like to see it published, but I have provided some notes, comments, and suggestions below.

I can imagine the amazing work who have been made to create this whole environment with all this embedded documentation. Unfortunately, I have the feeling that 1/ the use of an old Galaxy version and 2/ the manner to propose tutorials and tools to users will lead to an underutilization of the great potential of the approach and tools developed.

Article

I'm totally agree with a lot of statement of the article as considering the fact that "a new layer of computational methodology is needed" and I like the idea of "next generation tools" as the fact to consider first the biological question. Unfortunately, I found that the manuscript is quite difficult to read, for sure because some concept categorizations are quite hard to follow for someone like me (considering classes of multiplicity, algorithms or statistics), but maybe some improvement in the presentation of the concepts, better figures, schemas, can help the reader better catch and understand the presented complexity and associated challenges.

1) You mention p6 "in this example, using the Forbes measure to assess co-occurrence resulted in a biologically very reasonable ranking of potentially TFs, whereas the Jaccard measure produced a ranking that appeared severely biased by the number of peaks in each track from the suite". I'm not an expert of Forbes and Jaccard measure, but I'm interested to know more. Can you go further and explain more deeply this finding and give the reasons of this statement?

2) You mention p10 that GSuite HyperBrowser is prepared for future extensions in a variety of dimensions. Considering following test relative comments, and notably the problem associated to the

use of an old Galaxy instance, I'm quite afraid this flexibility will be hard to maintain. Can you argue on that point?

I'm totally agree with the statement about the fact that current statistics only relate to pure location data and I encourage authors to go ahead on the huge challenge behind this statement!

3) Concerning "consistent terminology for track metadata", can the authors give some insight, maybe regarding the use of ontology or web semantic approaches?

4) Figures appear to be in a quite bad quality, but maybe this is just because of the "review formatting"?

5) Reference 21 shows some "???"

Tests on the Genomic HyperBrowser instance

The Genomic HyperBrowser galaxy instance is reachable at https://hyperbrowser.uio.no/gsuite/ tested in a public mode (without account)

6) A first general comment is related to the fact to customize a Galaxy instance. If I really like the idea of having a personal dedicated instance with advanced functionalities and better graphics, to my knowledge, all deeply customized instance I have used, suffered many problems including update capabilities. I see that the Genomic Hyperbrowser is not based on a recent Galaxy instance (and maybe a quite very old one). This is maybe not totally due to this customization layer, but often, customization leads to a bigger cost related to instance update. This is really not a good thing when you work with a tool like Galaxy who is often updated and this can lead to security problems and difficulties to use wonderful new functionalities as Interactive tours mentioned above or the scratchbook view.

7) From the main page, I met difficulties to access screencasts from the "Brief demos" section. Apparently, the https://screencast.uninett.no/relay/ansatt/geirksauio.no/... Links were broken at the moment I want to access it.

Switching to the "Basic Mode" section, I really appreciated the use of the biological question as a starting point! I think this is something we have to use more generally when presenting bioinformatics tools functionalities.

8) Maybe the use of Galaxy "interactive tour" functionality (https://usegalaxy.org/tours) will be a more accurate manner to present GSuite concepts?

9) Executing the tool "Which tracks (in a suite) coincide most strongly with a separate single track?", outputs are "customhtml" or "gsuite" data types. Again, as the Galaxy instance is not up-to-date, I can understand the use of html content powered by Highcharts. But using html and moreover customhtml format to present a table and associated graph don't appear the best manner to do for me. I always

prefer to have the raw table, and here, at least, a possibility will be to propose the raw table in classical tabular format + an advanced html output. An alternative to your "customhtlm" output can be a report as for MultiQC (http://cloud-26.genouest.org/datasets/aa57f14b92934351/display/?preview=True). Concerning graphs, with an up-to-date Galaxy instance, it will be possible to generate it from the "Graph Visualization" functionality. Another way to have a dedicated visualization functionality can be the creation of a "real" Galaxy visualization (as for Trackster, Philoviz, …).

10) It would be good to have the scratchbook functionality to avoid escaping the "Basic Mode tuto page" each time you want to see a dataset.

After using Basic mode section related tuto, I have tested several tools as "Create a remote GSuite from a public repository" or "Convert GSuite tracks from remote to primary (Download the tracks to the server)".

11) Trying to view a hidden "GSuite track storage" dataset lead me to this strange message :

"This history element contains GSuite track data, and is hidden by default.

If you want to access the contents of this GSuite, please use the tool: DOES_NOT_EXIST_YET "

Is such a tool in development ?

12) You write :

"This example task can be seen as a concrete instance of the more general question "How does a query track overlap the different tracks of a suite?", where:

The query track is a dataset of genomic features (e.g. the genomic locations of specific SNP variants);

The suite of tracks (a GSuite instance) is a collection of related genomic feature datasets (e.g. a collection of datasets which map the areas of active chromatin, with each dataset describing the genomic regions of DNaseI accessibility for a separate cell type)."

Maybe pictures are a better way to illustrate the query tracks / suite of tracks concept ?

13) A little typo on Basic Mode / Analysis step / 1-c "supported file formats" : the hyperlink is not activated for entire sentence….

Level of Interest

Please indicate how interesting you found the manuscript: An article of importance in its field

Quality of Written English

Please indicate the quality of language in the manuscript: Acceptable

Declaration of Competing Interests

Please complete a declaration of competing interests, considering the following questions:

- Have you in the past five years received reimbursements, fees, funding, or salary from an organisation that may in any way gain or lose financially from the publication of this manuscript, either now or in the future?
- Do you hold any stocks or shares in an organisation that may in any way gain or lose financially from the publication of this manuscript, either now or in the future?
- Do you hold or are you currently applying for any patents relating to the content of the manuscript?
- Have you received reimbursements, fees, funding, or salary from an organization that holds or has applied for patents relating to the content of the manuscript?
- Do you have any other financial competing interests?
- Do you have any non-financial competing interests in relation to this paper?

If you can answer no to all of the above, write 'I declare that I have no competing interests' below. If your reply is yes to any, please give details below.

I declare that I have no competing interests

I agree to the open peer review policy of the journal. I understand that my name will be included on my report to the authors and, if the manuscript is accepted for publication, my named report including any attachments I upload will be posted on the website along with the authors' responses. I agree for my report to be made available under an Open Access Creative Commons CC-BY license (http://creativecommons.org/licenses/by/4.0/). I understand that any comments which I do not wish to be included in my named report can be included as confidential comments to the editors, which will not be published.

I agree to the open peer review policy of the journal
