# Supplementary material for: GSuite HyperBrowser: integrative analysis of dataset collections across the genome and epigenome
Source: Gigascience. 2017 Apr 27;6(7):1–12. doi: 10.1093/gigascience/gix032 (PMC5493745; doi:10.1093/gigascience/gix032)
Supplement: Reviewer-1-Report-(Revision-1).pdf [file gix032_Reviewer-1-Report-(Revision-1).pdf]

Reviewer Report

Title: "GSuite HyperBrowser: integrative analysis of dataset collections across the genome and epigenome"

Version: Revision 1 **Date:** 3/3/2017

Reviewer name: Yvan Le Bras

Reviewer Comments to Author:

Some comments below:

*In the "Classes of multiplicity for analyses of track suites" section, authors mentions figure3a, 3b ... instead of 4a, 4b, ... Moreover, on the Figure 4, there is no a), b), c) or d).

*Testing https://hyperbrowser.uio.no/hb/#!mode=basic (warning, as I have had very low internet connection, issues mentionned here are maybe due to this technical limitation and not to a Galaxy instance problem):

*In the basic mode section / - click here to load a sample track with Multiple Sclerosis-associated regions, expanded 10kb in both directions (you will be automatically redirected back to this page if you choose this option); Not automatically redirected to the mentionned page, but on the home page

*Same with 2b step - click here to load a sample GSuite of DNaseI accessibility for different cell types (you will be automatically redirected back to this page if you choose this option);

*As you are using "customhtml" output to propose to the user to export raw data table, it seems that user can't easily create a workflow using this output table. Maybe it can be of interest to propose directly on the tool formular an option for a "classical" text export.

Level of Interest

Please indicate how interesting you found the manuscript: An article of importance in its field

Quality of Written English

Please indicate the quality of language in the manuscript: Acceptable

Declaration of Competing Interests

Please complete a declaration of competing interests, considering the following questions:

- Have you in the past five years received reimbursements, fees, funding, or salary from an organisation that may in any way gain or lose financially from the publication of this manuscript, either now or in the future?

- Do you hold any stocks or shares in an organisation that may in any way gain or lose financially from the publication of this manuscript, either now or in the future?
- Do you hold or are you currently applying for any patents relating to the content of the manuscript?
- Have you received reimbursements, fees, funding, or salary from an organization that holds or has applied for patents relating to the content of the manuscript?
- Do you have any other financial competing interests?
- Do you have any non-financial competing interests in relation to this paper?

If you can answer no to all of the above, write 'I declare that I have no competing interests' below. If your reply is yes to any, please give details below.

I declare that I have no competing interests

I agree to the open peer review policy of the journal. I understand that my name will be included on my report to the authors and, if the manuscript is accepted for publication, my named report including any attachments I upload will be posted on the website along with the authors' responses. I agree for my report to be made available under an Open Access Creative Commons CC-BY license (http://creativecommons.org/licenses/by/4.0/). I understand that any comments which I do not wish to be included in my named report can be included as confidential comments to the editors, which will not be published.

I agree to the open peer review policy of the journal
