# Supplementary material for: GSuite HyperBrowser: integrative analysis of dataset collections across the genome and epigenome
Source: Gigascience. 2017 Apr 27;6(7):1–12. doi: 10.1093/gigascience/gix032 (PMC5493745; doi:10.1093/gigascience/gix032)
Supplement: Reviewer-2-Report-(Original-Submission).pdf [file gix032_Reviewer-2-Report-(Original-Submission).pdf]

Reviewer Report

Title: "GSuite HyperBrowser: integrative analysis of dataset collections across the genome and epigenome"

Version: Original Submission **Date:** 10/17/2016

Reviewer name: Eric Rasche

Reviewer Comments to Author:

This paper covers a comprehensive tool suite useful in the investigation of and comparison of track based datasets. They have produced a large set of inter-operable tools, provided what look to be quite valid experimental use cases in situations where the current landscape was lacking. They then showed that their new tools neatly solved the problems.

Most of my comments will be on the technological side, as I am somewhat less qualified to speak on the data analysis portion.

Remote vs Local

It is nice to see the abstractions over local vs remote data sources. However it seems that a number of tools (basically all tools?) only function on local data sources breaking that transparency. Exporting tracks is an example of this, it seems to just select a single line from a GSuite file, but due to the addition of features to that tool (previewing, format conversion) this cannot be abstracted to remote data sources.

The sentence "can represent data at a local or remote server" got me a bit excited for a format which behaves transparently despite data locality, but I recognise the rationale behind the implementation. Re-reading this paragraph, it seems this was just me jumping to conclusions. Some of these tools could perhaps be extended to work on both local and remote datasets with greater flexibility, but I would not say this is important for the publication of this paper.

Proto-Galaxy

I remember the presentation at GCC, proto galaxy for tool development is quite interesting as a concept. For actually deploying these tools to local galaxies, however, it comes across as a negative that we need a specific, old version of galaxy to run these tools. The fact that these tools are not available as standard tools with galaxy XML tool definitions will significantly hinder adoption by the wider community.

Availability of Sources

The authors have done a good job of making their work accessing on public repositories. It is really wonderful to see this!

Unfortunately it seems that the SVN repos listed in both the INSTALL.txt file provided and the downloads page (https://hyperbrowser.uio.no/hb/static/download.html) reject my access. (svn: E000111: Can't connect to host 'invitro.titan.uio.no': Connection refused) I would recommend fixing this before publication.

Additionally the INSTALL.txt does not mention the need to create a config/LocalOSConfig.py file, this should be adjusted for clarity.

Assorted Comments / Issues

The basic vs advanced mode is quite useful, especially for someone unfamiliar with the tools installed in your system. Having a short list of pre-configured analyses is a great thing.

Many of the features implemented by GSuite files also exist in more recent releases of Galaxy as dataset collections. The authors should consider replacing future versions of GSuite files with a set of custom datatypes supporting the same metadata, and taking advantage of the new collections to make comparisons within and across lists of files.

The second supplementary file lists two URLs that are not rendered properly: the three dashes have been converted to an emdash preventing easy access.

Level of Interest

Please indicate how interesting you found the manuscript: An article of importance in its field

Quality of Written English

Please indicate the quality of language in the manuscript: Acceptable

Declaration of Competing Interests

Please complete a declaration of competing interests, considering the following questions:

- Have you in the past five years received reimbursements, fees, funding, or salary from an organisation that may in any way gain or lose financially from the publication of this manuscript, either now or in the future?
- Do you hold any stocks or shares in an organisation that may in any way gain or lose financially from the publication of this manuscript, either now or in the future?
- Do you hold or are you currently applying for any patents relating to the content of the manuscript?
- Have you received reimbursements, fees, funding, or salary from an organization that holds or has applied for patents relating to the content of the manuscript?
- Do you have any other financial competing interests?
- Do you have any non-financial competing interests in relation to this paper?

If you can answer no to all of the above, write 'I declare that I have no competing interests' below. If your reply is yes to any, please give details below.

I declare that I have no competing interests.

I agree to the open peer review policy of the journal. I understand that my name will be included on my report to the authors and, if the manuscript is accepted for publication, my named report including any attachments I upload will be posted on the website along with the authors' responses. I agree for my report to be made available under an Open Access Creative Commons CC-BY license (http://creativecommons.org/licenses/by/4.0/). I understand that any comments which I do not wish to be included in my named report can be included as confidential comments to the editors, which will not be published.

I agree to the open peer review policy of the journal
