# Supplementary material for: GSuite HyperBrowser: integrative analysis of dataset collections across the genome and epigenome
Source: Gigascience. 2017 Apr 27;6(7):1–12. doi: 10.1093/gigascience/gix032 (PMC5493745; doi:10.1093/gigascience/gix032)
Supplement: Reviewer-3-Report-(Original-Submission).pdf [file gix032_Reviewer-3-Report-(Original-Submission).pdf]

Reviewer Report

Title: "GSuite HyperBrowser: integrative analysis of dataset collections across the genome and epigenome"

Version: Original Submission **Date:** 3/18/2017

Reviewer name: Anil Thanki

Reviewer Comments to Author:

GSuite HyperBrowser is very good and well-thought piece of software. It's integration into Galaxy makes it more usable for many users from various field. As I am not expert in Statistical analysis, I am writing from computational side of this paper.

The manuscript is well written and explains useful use-cases and workflow, it looks like it lives up to the claims authors make in this article. I tried running some examples, shown on the website, and they run smoothly.

I noted GSuite uses column based text file for metadata, of which I am not against, but It could be restrictive in case of adding extra information, this issue could be easily handled using JSON format.

Also, it would be better if authors mentioned about visual bits of the software in manuscript, and some visual representation of statistical analytics would be a great addition.

I noticed the file format for the output was unknown in some steps (GSuite - remote files, and while creating GSuite), when following example steps, it could be misleading for some users, but not a deal breaker.

Though GSuite HyperBrowser is available on GitHub, but link is not clearly mentioned in manuscript.

In my opinion the work represents a very good effort by authors to make their work easily accessible to users, by the Galaxy community, hence my suggestion to its publication.

Level of Interest

Please indicate how interesting you found the manuscript: An article whose findings are important to those with closely related research interests

Quality of Written English

Please indicate the quality of language in the manuscript: Acceptable

Declaration of Competing Interests

Please complete a declaration of competing interests, considering the following questions:

- Have you in the past five years received reimbursements, fees, funding, or salary from an organisation that may in any way gain or lose financially from the publication of this manuscript, either now or in the future?
- Do you hold any stocks or shares in an organisation that may in any way gain or lose financially from the publication of this manuscript, either now or in the future?
- Do you hold or are you currently applying for any patents relating to the content of the manuscript?
- Have you received reimbursements, fees, funding, or salary from an organization that holds or has applied for patents relating to the content of the manuscript?
- Do you have any other financial competing interests?
- Do you have any non-financial competing interests in relation to this paper?

If you can answer no to all of the above, write 'I declare that I have no competing interests' below. If your reply is yes to any, please give details below.

I declare that I have no competing interest

I agree to the open peer review policy of the journal. I understand that my name will be included on my report to the authors and, if the manuscript is accepted for publication, my named report including any attachments I upload will be posted on the website along with the authors' responses. I agree for my report to be made available under an Open Access Creative Commons CC-BY license (http://creativecommons.org/licenses/by/4.0/). I understand that any comments which I do not wish to be included in my named report can be included as confidential comments to the editors, which will not be published.

I agree to the open peer review policy of the journal
